# Wearables for running gait analysis: A study protocol

**DOI:** 10.1371/journal.pone.0291289

**Published:** 2023-09-11

**Authors:** Rachel Mason, Alan Godfrey, Gillian Barry, Samuel Stuart

**Affiliations:** 1 Department of Sport, Exercise and Rehabilitation, Northumbria University, Newcastle, United Kingdom; 2 Department of Computer and Information Sciences, Northumbria University, Newcastle, United Kingdom; 3 Northumbria Healthcare NHS foundation trust, North Shields, United Kingdom; 4 Department of Neurology, Oregon Health and Science University, Portland, Oregon, United States of America; Professionshøjskolen UCN: Professionshojskolen UCN, DENMARK

## Abstract

Quantitative running gait analysis is an important tool that provides beneficial outcomes to injury risk/recovery or performance assessment. Wearable devices have allowed running gait to be evaluated in any environment (i.e., laboratory or real-world settings), yet there are a plethora of different grades of devices (i.e., research-grade, commercial, or novel multi-modal) available with little information to make informed decisions on selection. This paper outlines a protocol that will examine different grades of wearables for running gait analysis in healthy individuals. Specifically, this pilot study will: 1) examine analytical validity and reliability of wearables (research-grade, commercial, high-end multimodal) within a controlled laboratory setting; 2) examine analytical validation of different grades of wearables in a real-world setting, and 3) explore clinical validation and usability of wearables for running gait analysis (e.g., injury history (previously injured, never injured), performance level (novice, elite) and relationship to meaningful outcomes). The different grades of wearable include: (1) A research-grade device, the Ax6 consists of a configurable tri-axial accelerometer and tri-axial gyroscope with variable sampling capabilities; (2) attainable (low-grade) commercial with proprietary software, the DorsaVi ViMove2 consisting of two, non-configurable IMUs modules, with a fixed sampling rate and (3) novel multimodal high-end system, the DANU Sports System that is a pair of textile socks, that contain silicone based capacitive pressure sensors, and configurable IMU modules with variable sampling rates.

**Clinical trial registration: Trial registration**: NCT05277181.

## Introduction

Running is one of the most popular sport and recreational activities worldwide, as well as being a core component to many sports [1]. With the growing popularity of running, a concurrent increase in the incidence of running related injuries has also been reported [2, 3]. It is well established that an abnormal running gait is a contributing factor to running related injuries [4]. Quantitative running gait analysis, as a (clinical) tool for minimising injury risk and as a performance measure, has been well documented in the literature [5–7], with improvements in running gait, leading to maximized mechanical efficiency thus enhancing performance, and reducing injury risk [8].

A variety of methods exist to examine running gait, in low-resource settings practitioners have largely been limited to the use of subjective clinical observations using rating scales (e.g., The High-Level Mobility and Assessment tool), and/or 2D video analysis, which may not be sensitive to subtle changes in performance with training or injury [9–11]. To analyse less readily identifiable outcomes, such as fine motor movement in spatiotemporal characteristics (e.g., stride length, stride time, cadence, speed), kinematic (e.g., angular velocity and joint angles) and kinetic (e.g., ground reaction forces, GRF), more cumbersome and expensive traditional (reference/gold-standard) gait laboratory equipment are required (e.g., 3D motion capture, force plate equipment, instrumented treadmills). Equally, use of laboratories for running gait assessment is further limited due to the need for trained practitioners to collect and analyse data and requirement to attend a bespoke setting. Furthermore, traditional lab-based approaches provide limited understanding of running from ‘real-world’ environments [12–14] and often use constrained protocols that may not represent usual running behaviour, such as unnatural force platform targeting [15]. Numerous studies have sought to overcome this issue by using instrumented treadmills, however, further studies demonstrate inconsistencies in running gait between over-ground and treadmill running [16]. In order to enhance understanding of running gait, further research in a natural running environment is required [15].

Wearable devices offer an alternative to overcome traditional assessment limitations and are becoming increasingly popular amongst runners, coaches and clinicians [17]. Wearables using accelerometers, gyroscopes, and magnetometers, applied individually or in combination as an inertial measurement unit (IMU), and ‘pressure-sensitive’ insoles enable examination of a combination of spatiotemporal, kinetic, and kinematic variables and have become a viable alternative due to their portability and affordability [18]. Wearables can quantify running gait outcomes in any setting (i.e., laboratory, outdoor/real-world), and have the potential to be an effective tool to capture the full duration and nuances of a run, which ultimately may enhance understanding of running performance, fatigue, and injury mechanisms [19, 20].

Wearables have seen an expansive increase in personal use, as well as application in industry and research. However, there is a lack of evidence for validity, reliability, and application of wearables for running gait assessment [19–22], with different grades of devices receiving different levels of testing. For example, many commercial devices receive little / no validation testing before commercial launch, whereas research-grade devices that are developed by academic researchers tend to receive validation testing but may not be applied within relevant populations to inform clinical or performance outcomes [18–22]. Therefore, this pilot study aims to examine the validity, reliability, and application of various grades of wearable devices (research-grade, commercially available and novel multi-modal) for running gait assessment. The presented protocol will follow an evidence-based approach to drive the appropriate adoption of fit-for-purpose wearable technologies [23]. An evaluation of the usefulness and utility is only applicable after gaining evidence and assurance that the underlying data obtain valid and reliable results to answer a given question [23].

### Analytical validation aims

1. ***Laboratory validation***: Determine the concurrent validity and test re-test reliability of wearables (research-grade, commercial and novel multimodal devices) for running gait analysis against gold-standard reference measures in adult runners.
2. ***Real-world validation***: Investigate concurrent validity of real-world running gait as measured by different wearables (research-grade, commercial and novel multimodal devices) in adult runners.

### Exploratory clinical validation aims

1. Explore differences in running gait outcomes within different clinical (injured vs non-injured) and performance (elite vs novice) groups.
2. Explore relationships between real-world running gait and demographic characteristics (e.g., sex, age) in different clinical and performance groups (e.g., those with a previous injury or different performance level).
3. Investigate the usability of the wearable devices in adult runners.

## Materials and methods

### Study design

**Fig 1:**
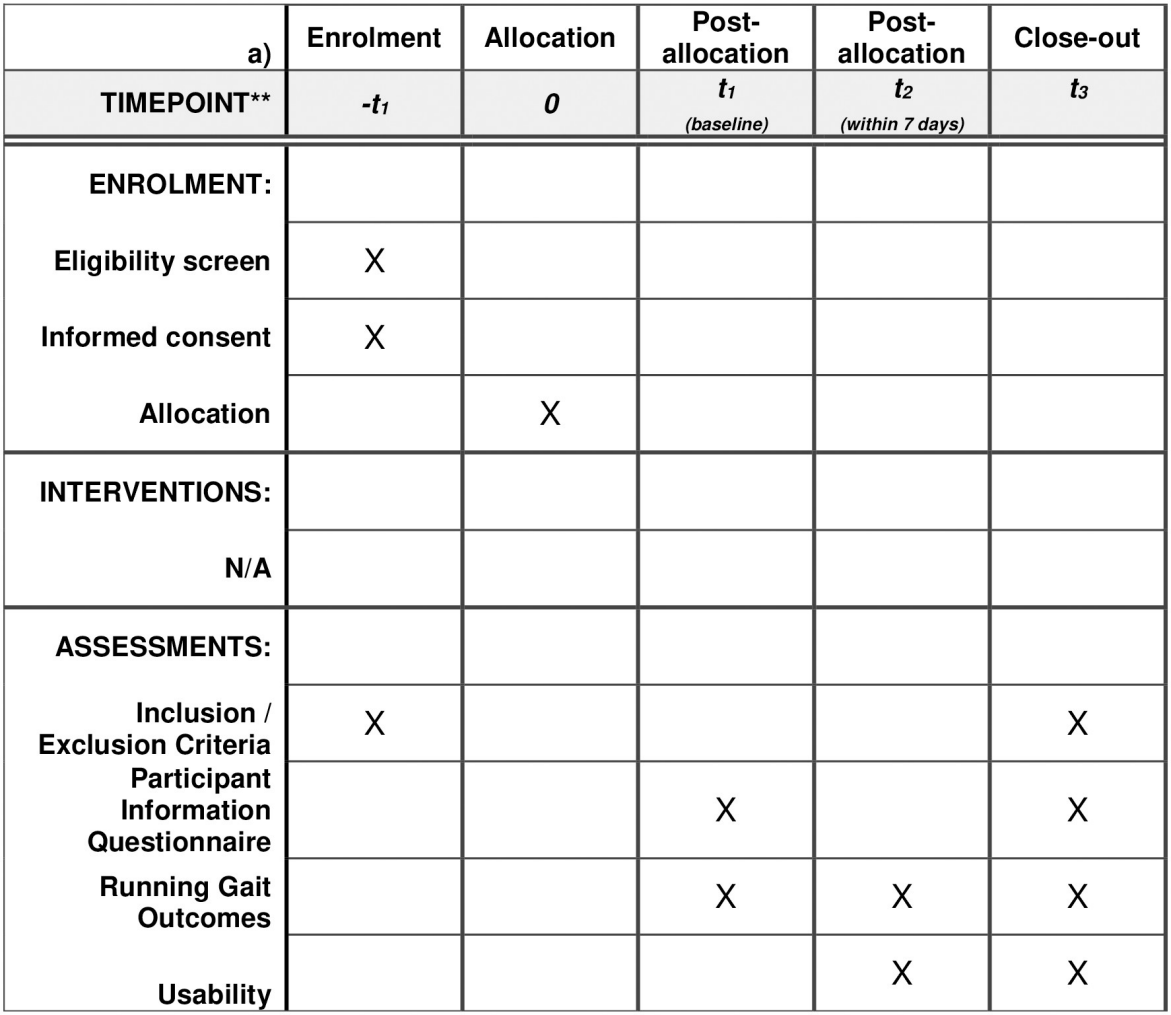
SPIRIT diagram. The overall schedule and time commitment for study participants for both a) laboratory testing and b) real-world environment testing.

This is a cross-sectional study that will assess the validity and reliability of three grades of wearables (research, entry-level commercial and advanced / novel multimodal). The protocol was developed according to the Standard Protocol Items: Recommendations for Interventional Trials’ (SPIRIT) checklist [24], as appropriate (see S1 File). As this is a study protocol, no data has been included and conforms to PLOS data policy. The overall schedule and time commitment for trial participants are depicted in Fig 1 and S1 File.

### Ethical approval and trial registration

A Northumbria University research ethics committee granted ethical approval (Ref: 21603 and 33358) and this study conforms to the Declaration of Helsinki. The trial has been reviewed and registered with Clinicaltrials.gov (NCT05277181).

### Participants

A total of 80 runners with a variety of training experience will be recruited and assessed within laboratory (n = 40) and real-world environments (n = 40). The common inclusion and exclusion criteria are outlined in Table 1.

**Table 1:** Participant criteria.

Inclusion Criteria	Exclusion Criteria
Aged 18–70 years.	Medical history of disability that affects running gait safety or ability to follow instructions/tasks.
English as a first language or fluency.	
Able to run 5km without stopping.	Known illness or disease that would prevent their participation in strenuous physical activities (e.g., cardio-respiratory conditions or acute COVID-19).
Take part in running of some form at least twice per week (e.g., 5km run).	
	If the participant is unable to comply with the testing protocol, they will not be recruited.

#### Recruitment

An advertisement poster and email text invitation will be sent via email to Northumbria University running clubs and running clubs within England. Recipients will be asked to pass on the recruitment poster to potential interested parties (i.e., family or friends). Recruitment began in January 2022. Those interested will then be given a participant information sheet and a letter concerning the study and consent. Written, informed consent will be collected from all participants.

### Equipment

#### Reference standard: 3D motion analysis

A 14-camera motion analysis system (VICON, Oxford, UK), sampling at 250Hz will be used. Calibration of the Vicon system will be conducted before each data collection. Sixteen reflective markers will be placed on the participants lower limb before testing, and a static calibration trial will be initially collected to form a musculoskeletal model [25]. Using a small amount of double-sided tape, the place of markers will be attached bilaterally to the following landmarks: anterior superior iliac spine, posterior superior iliac spine, mid-lateral thigh, lateral knee joint line, lateral mid-shank, lateral malleoli, calcaneal tuberosity, and base of the second metatarsal.

**Fig 2:**
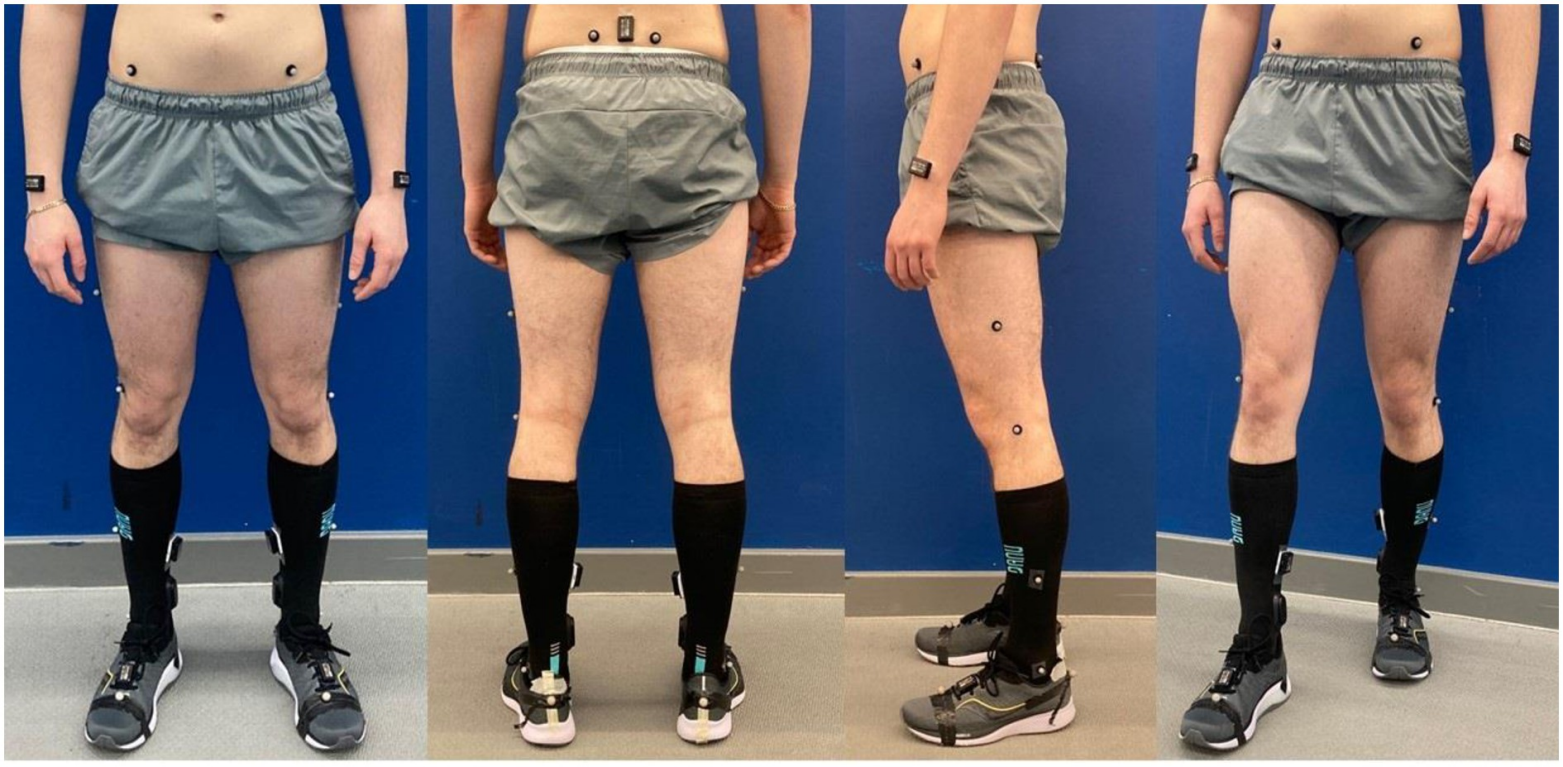
Participants’ setup. The positions for the three grades of wearables and reflective markers for 3D motion capture are shown.

The participants’ specific information of weight, height, ankle width, knee width, and leg length (from posterior iliac spine to medial malleolus) will be measured in the lower body model [26]. The Plug-in-Gait (PiG) lower body model will be used to analyse movement at the joints and evaluate all parameters. The lower body will be modelled as seven segments (one pelvis, two thighs, two shanks, and two feet). A normal gait cycle will be defined from the initial heel-to-heel contact with the same limb. Additional information of the PiG calculations can be found on Vicon’s website. Fig 2 shows the participants’ setup of the anterior, lateral, and posterior views with the markers in place.

#### Reference standard: Force plate system

Two staggered 0.5-m-long force plates (AMTI, Watertown, MA, USA), sampling at 1000 Hz will be embedded in the middle of a 15m walkway.

#### Wearables

With many wearables available, it is difficult to choose the most suitable [21]. Accordingly, this protocol includes options from three categories to suit varying user needs: (1) modifiable research-grade; (2) attainable (low-grade) commercial with proprietary software; and (3) novel multimodal high-end system [18]. Accordingly, the three wearable types presented here are: research grade AX6 (Axivity, https://axivity.com/), entry-level commercial DorsaVi ViMove2 (DorsaVi, www.dorsavi.com/vimove/) and advanced multimodal DANU System (DANU Sports, www.danusports.com/).

Typically, research-grade devices require users to have specialist / technical knowledge due to the tendency of devices to provide the raw data only. Therefore, the user often needs to create and implement algorithms to achieve tangible outcomes. However, these devices offer the flexibility to define wear location and creation of novel outcomes [27]. Consumer level / commercial wearables with proprietary software are sought to be user-friendly, requiring little technical knowledge or experience, producing outcomes automatically. For these devices, it is often unclear how reference limits are set, or established for such functions [27]. Rather than showing the raw data collected by the wearable, users typically export information after it has been analysed and filtered through an algorithm. More advanced multi-modal devices (e.g., combined IMU and pressure sensors in a single system) offer numerous sensing capabilities with multiple algorithms that are enabling laboratory grade sensing in any location. Accordingly, the integrated sensor arrangement enables a comprehensive assessment of running gait (i.e., spatiotemporal, and kinetic outcomes) and attempt to offer the advantages of both research-grade and commercial wearables.

**Fig 3:**
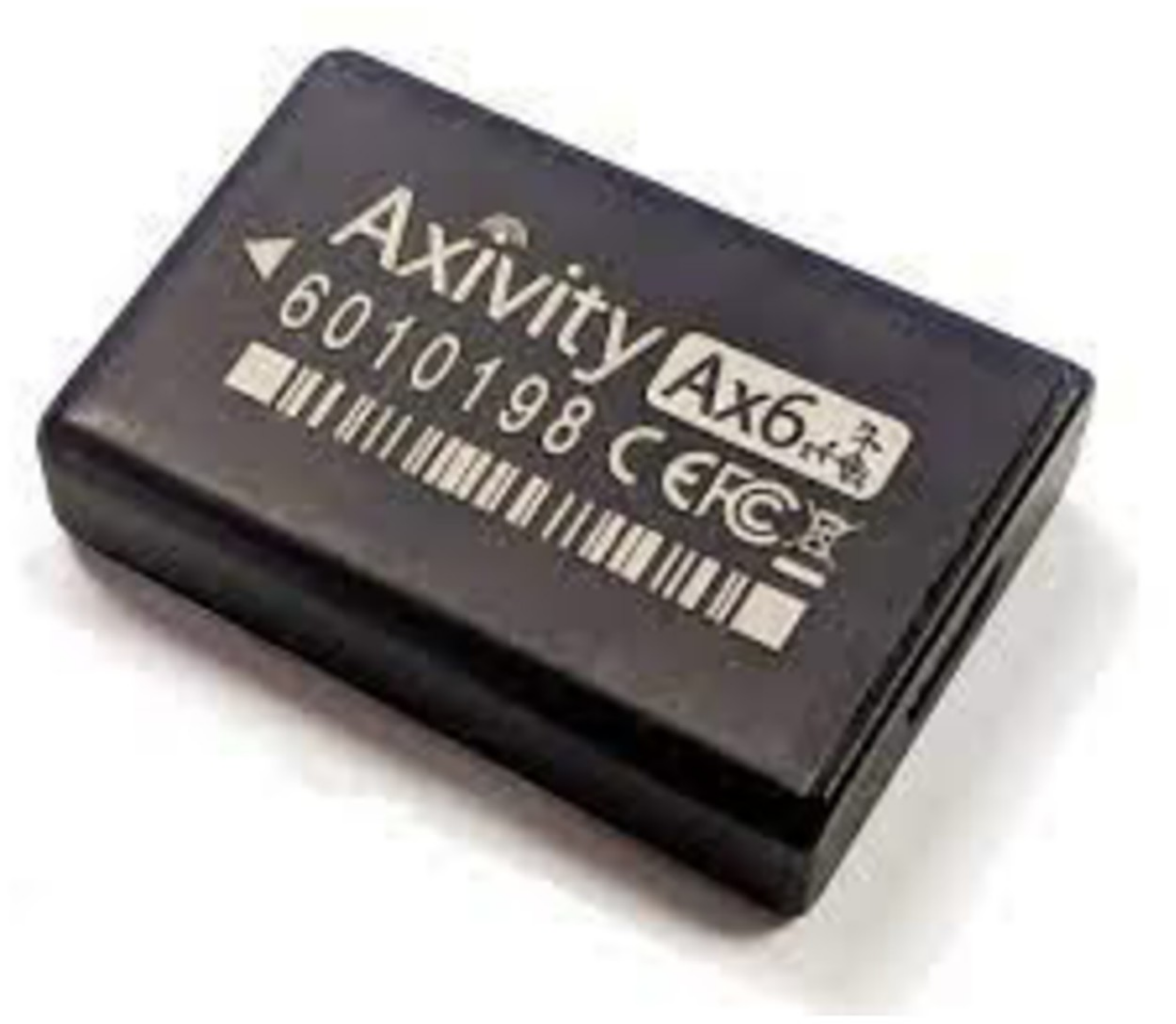
Axivity AX6.

*Axivity AX6*. The AX6 (2.3 × 3.3 × 0.8 cm, 11g) consists of a configurable tri-axial accelerometer (±2, ±4, ±8, ±16g) and tri-axial gyroscope (±125 –±2000°/s) with variable sampling capabilities (12.5Hz - 1600Hz), set via a configuration proprietary software (OmGUI). Here, the device will be configured to a sampling rate of 200Hz, ±16g accelerometer and ±2000°/s gyroscope. Although it can be worn on any anatomical landmark, due to lack of proprietary analytical software, it will be secured onto the talus joint of each shoe with medical tape, Fig 3. The AX6 is considered a valid technology which can capture data in controlled and free-living environments [28]. Data are recorded and stored locally on the device and download via OmGUI upon completion of recording.

**Fig 4:**
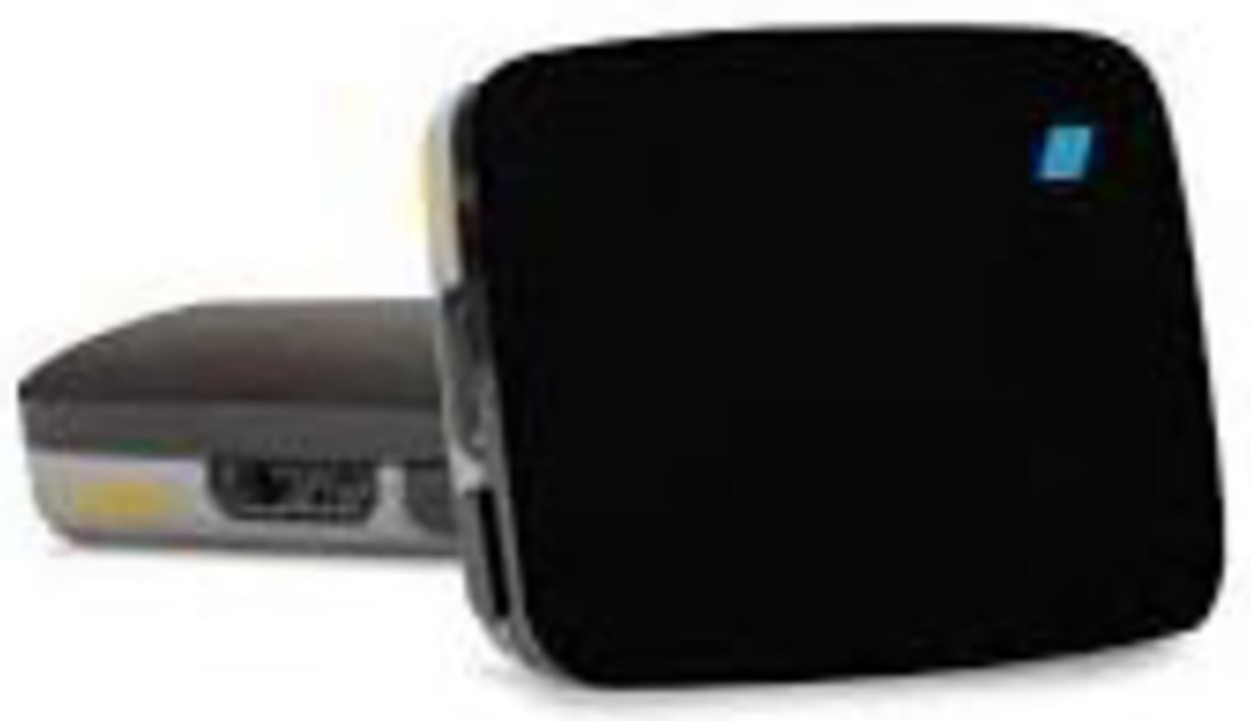
DorsaVi ViMove2.

*DorsaVi ViMove2*. The DorsaVi ViMove2 consists of two IMUs modules, each comprising a tri-axial accelerometer (±16g) with tri-axial magnetometer and tri-axial gyroscope (±250°/s), sampling at 100Hz. Modules will be placed onto the anterior surface of the mid-shank of the right and left tibia, as per manufacturer’s guidelines (Fig 4) [29]. Tibia sensor placement is based on the anthropometric measurement of height and a ruler is used to measure exact placement, which is the midpoint between the knee and ankle along the anterior surface of the tibia [30]. After placement of bilateral tibia sensors, the DorsaVi ViMove2 is calibrated via its ViPerform software. Data are transmitted through wireless channel to a recording and feedback device, from which data can be offloaded onto PC for further analysis.

**Fig 5:**
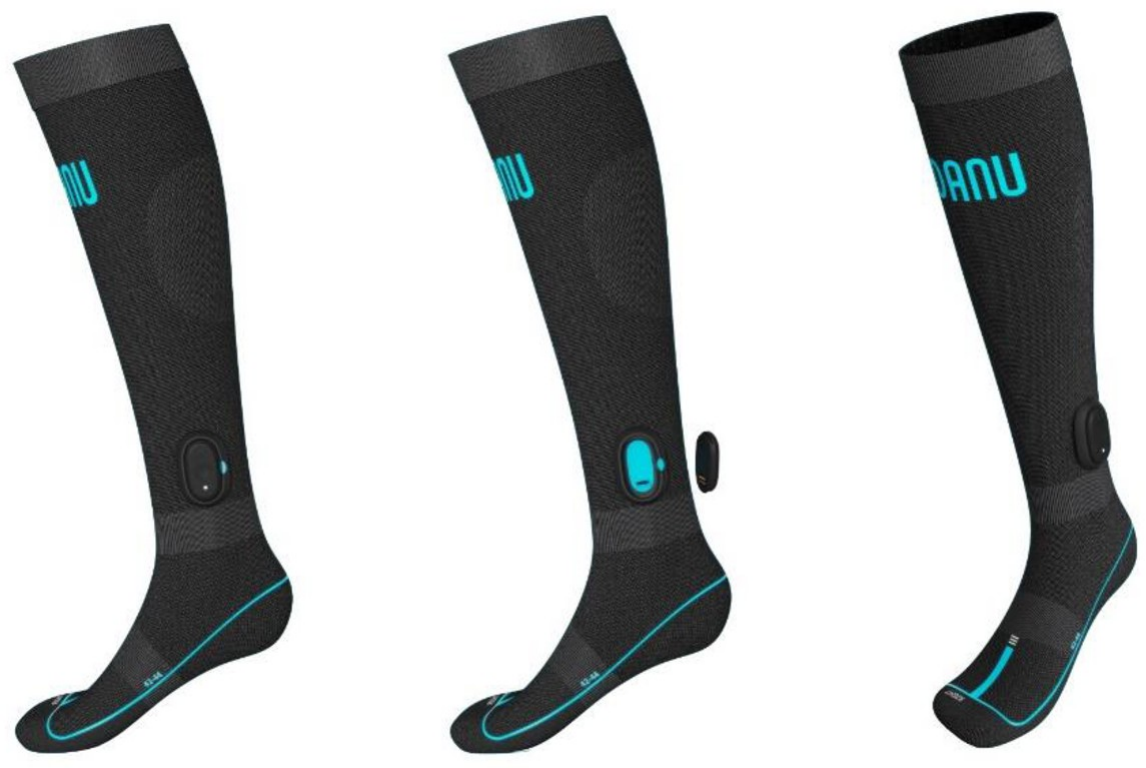
DANU sports system.

*DANU sports system*. The DANU Sports System (Fig 5) is a pair of textile socks, to be worn on both feet. Each sock contains 15 silicone based capacitive pressure sensors, and an IMU module attached onto the anterior surface of the mid-shank of tibia. Each IMU module is Bluetooth enabled for data transmission and is comprised of two configurable tri-axial accelerometers (Accelerometer 1 ±2g, ±16g, and ±32g, Accelerometer 2 ±50g, ±100g, and ±200g), gyroscope (±2000°/s), magnetometer, and with variable sampling rates (60 – 250Hz). The IMU module includes in-built memory for data collection. For this protocol, the DANU Sports System will be configured to a default sampling rate of 250Hz, ±16g, ±50g accelerometers and ±2000°/s gyroscope. Data is collected via Bluetooth on Apple devices 2018 or later (iPad or iPhone devices are required to have at least Bluetooth 5.0 connectivity), and data processing is run through a custom-made Apple application for real-time feedback and visualisation.

### Data collection

Prior to data collection, all participants regardless of study arm (laboratory or real-world) will complete a questionnaire to provide information pertaining to their demographics, lifetime athletic injury history, medical history, sporting pursuits and running personal bests (S1 Appendix). Injury will be classified as “any muscle, bone, tendon or ligament pain in the lower back/legs/knee/foot/ankle that caused a restriction or stoppage of running (distance, speed, duration or training) for at least 7 days or 3 consecutive scheduled training sessions, or that required the runner to consult a physician or other health professional” [31–33].

#### Laboratory testing

Laboratory testing will be conducted at the biomechanics laboratory Northumbria University, Newcastle upon Tyne, United Kingdom. Within the controlled laboratory environment, we will conduct a concurrent data collection with the multimodal, commercial, and research-grade wearables to determine their validation and reliability for running gait analysis compared to laboratory reference standards (3D motion capture and force plates). Recruitment for the laboratory testing began in September 2021 with an expected end date September 2023.

*Participants*. Within the laboratory testing 40 individuals will be recruited, specifically 20 with and 20 without a history of previous lower limb injury (but fully recovered at time of assessment and able to run safely, in line with inclusion / exclusion criteria).

*Laboratory reference set-up*. As previously described, a 3D motion capture and force plate system will be used as the ‘gold-standard’ reference measures.

*Running procedures*. All participants will run overground and, on a treadmill, (Spirit fitness XT485). Participants will be provided with a standardised, neutral cushioning running shoe (Saucony Guide Runner) to wear during testing in order to remove bias [34]. Prior to testing, participants will run on the treadmill at a comfortable speed for a warm-up and to familiarise themselves with running on a treadmill. Participants will be allowed breaks at any point or permitted to abort the trial at any time. For the overground segment, participants will run at a comfortable self-selected speed overground for 10m intermittently, where they will be asked to foot-strike two staggered force plates in the middle of the run (~5m point). Practice trials will be performed prior to data collection to allow participants to adjust their start position to strike the force plates correctly. A total of five successful recordings for each of the left and right leg will be captured. For the treadmill segment, participants will be asked to run at five speeds, four of these speeds will be standardised (i.e., 8, 10, 12 and 14 km/hr) and one speed will be their self-selected speed. Self-selected speed will be determined by the participant’s 5km personal best. The order of speed will be consistent across participants, starting at the slowest speed and progressing to the fastest to ensure the safety of participants. Data will be collected for 60s at each speed.

*Initial data processing*. Data will be collected simultaneously by the measurement systems, at the commencement and conclusion of each task, the time will be synchronized with a laptop and recorded by the assessor (i.e., time-stamp extraction). At the end of each testing session, the wearable and reference device data will be download onto a computer and stored in secure servers for further data processing and analysis.

#### Reliability testing

Each participant will complete the protocol in the same format in a repeated-measures design, approximately one week after the first session to establish the test-retest reliability.

#### Real-World environment testing

Data from the novel multimodal, commercial, and research-grade wearables will be collected within real-world environments to test the clinical / performance validity of the wearables (i.e., can they differentiate or provide meaningful data on relevant populations). Recruitment for the real-world environment testing began in June 2023 with an expected end date October 2023.

*Participants*. Within the real-world environment testing 40 individuals, specifically novice/amateur (n = 20) and expert/sub-elite (n = 20) running performance level. Performance level will be based on their 5km running time (i.e., age graded performance % [35]).

*Wearable location*. Each participant will be equipped with the DANU Sports System (socks on both feet), two Axivity AX6 sensors attached to the shoelaces and two DorsaVi ViMove2 sensors on the tibia.

*Running procedures*. All participants, regardless of performance level, will be asked to complete a 5km run on a standardised route in North-East England. The course will be run on a mixture of trail paths and concrete paths, with 174ft elevation gain throughout. Information about conditions (e.g., environment and shoes) and objective load data (e.g., time and pace) will be collected. Participants will wear their own running shoes.

#### System usability scale

Upon immediate completion of the real-world environment testing, participants will be asked to complete the System Usability Scale (SUS) to assess the usability of each of the wearables. The SUS is a simple scale based, multiple-choice questionnaire proposed by John Brooke in 1986 and has been widely adopted in product usability evaluations [36–38]. A final score is provided with interpretation based on a well-established reference standard, with an excellent reliability (0.85) [39]. Overall, the SUS is a quick and simple method for usability evaluation.

### Outcome measures

The primary outcome measures for this study are running gait outcomes as measured by the wearables and are described in Table 2. A secondary outcome measures is usability. Usability refers to the effectiveness, efficiency, and user satisfaction rating of a product (various grades of wearables) in an particular environment by a user for a specific purpose (running gait analysis) [40].

**Table 2:** The primary outcomes measured by each grade of wearable.

Outcome	Definition	Axivity AX6	DorsaVi ViMove2	DANU Sports System	Reference Measure
***Spatiotemporal***					
Ground Contact Time	The time between initial foot contact and toe-off for the same foot	**✓**	**✓**	**✓**	Force plate (overground) and 3D Motion Capture (treadmill)
Step Frequency	The number of steps taken during a given time	**✓**	**✓**	**✓**	3D Motion capture
Step Length	The distance between successive points of initial contact of the opposite foot	X	X	**✓**	3D Motion capture
Step Time	The time between two consecutive heel strikes of the opposite foot	**✓**	X	**✓**	Force plate (overground) and 3D Motion Capture (treadmill)
Foot-Strike Pattern	The way or angle when the foot first contacts the ground	**✓**	X	**✓**	3D Motion capture
Flight Time	The time between toe-off from one foot to initial contact of the other foot	**✓**	X	**✓**	Force plate (overground) and 3D Motion Capture (treadmill)
Symmetry	Any measure of imbalance between the right and left leg	**✓**	**✓**	**✓**	3D Motion capture
***Kinetic***					
Tibial Acceleration	Acceleration of the tibia, including peak positive acceleration, gradient, slope, magnitude, loading rate etc.	X	**✓**	**✓**	3D Motion capture

### Sample size calculation

This is the first study (pilot study) to use the novel multi-modal wearable system (DANU system) for gait analysis in any population, alongside other wearables and gold-standard measures. As such, there are few specific previous examples were available to guide estimates for sample size. Previous research that has shown statistically significant outcomes when investigating wearables for running gait assessment in sport have had sample sizes ranging from 1 [41] to 187 [42], with the average number of participants at 26 [21]. Thus, a sample size of 40 participants for analytical validation (i.e., comparison to gold-standards) is feasible and adequately powered [43, 44].

Regarding the exploratory clinical validation analysis, a sample size of 20 participants per group for the performance and clinical group analysis is adequate in this initial exploratory pilot study. While there are no specific rules for sample size calculations in pilot / initial exploratory studies, evidence suggests that 10–12 participants per group may be adequate, and that 20–30 participants per group would be enough to provide valid evidence to draw scientific conclusions [43–46]. Further to this, evidence suggests that to capture 85–90% of use issues 15–20 people per group are required for usability studies [46], therefore our sample of 20 participants per group should be adequate for this initial analysis. Following from the present pilot study, future research can calculate more specific sample sizes and control for relevant variables based on the presented findings.

### Data processing

All collected data will be anonymised, stored, and saved in the main computer, password protected. Data processing will be performed in Vicon Nexus. All markers will be labelled, and marker trajectories were filtered using a fourth order low-pass Butterworth filter via dynamic plug-in gait model with 6 Hz cut-off frequency. Gait identification will be achieved through visual inspection of foot strike and toe off for consecutive strides over the 60s. A threshold of 20 N in ground reaction force will used to determine initial contact and toe-off. Kinetic variables will be normalized using base factors of gravitational constant g (9.81 m/s^2^), leg length, L (m), and body mass, M (kg).

Wearable device data (DANU Sport and DorsaVi ViMove2) will be processed as per the manufacturer’s guidelines, whereas the Axivity AX6 data processing will be carried out from the nine axis (accelerometer, magnetometer, and gyroscope) raw signal by a previously validated in-house algorithm [47]. A master data table will be created with all available outcomes, categorised by subject and date, including a column to indicate distinguishing factors (e.g., those with previous injury history, different performance levels).

### Statistical analysis

#### Analysis common to all studies

The statistical analysis will be performed using statistical software SPSS 25 (SPSS Inc., Chicago, Illinois, USA). Demographic characteristics will be summarised using descriptive statistics, including mean, standard deviation, median, minimum, maximum, and inter-quartile range for continuous or ordinal data and percentages for categorical data. The descriptive statistics will be tabulated and presented graphically for clarity. All data will be checked for normality distributed with Shapiro-Wilks tests before conducting parametric tests. Non-normal continuous distributions will be transformed where appropriate to meet the requirements of parametric tests; otherwise, the equivalent non-parametric tests will be adopted. Data will also be assessed graphically (such as histograms or scatter plots) for clarity of information. Statistical significance will be determined at p < 0.05 unless otherwise stated. Due to the experimental nature and novelty of the work and wearable systems, we will not control for multiple comparisons in order to avoid a Type II error.

*Analytical validation analysis*. To examine analytical (concurrent) validation of the wearable devices (research-grade, commercial, novel multimodal) for running gait measurement, we will compare outcomes to relevant reference measures (i.e., force plates for ground contact time) in all laboratory testing participants (n = 40). To compare running gait outcomes from the wearables and reference systems, the absolute difference, Pearson’s correlation (r), intraclass correlations (ICC), the level of agreement through the Bland Altman plots, and 95% Limits of agreement (LoA) will be calculated [48]. Accuracy will be evaluated in terms of root mean squared errors (RMSEs).

To determine test re-test reliability of the wearables, r, and ICCs between the two testing time-points will be calculated [49]. A predefined ICC performance scale will be used, defined as poor (<0.50), moderate (0.50–0.75), good (0.75–0.90) or excellent (>0.90) [50]. ICC is a common statistic for evaluating repeatability and is needed to calculate the minimally detectable change (MDC) [51, 52]. The standard error of measurement (SEM) and standard deviation (SD) will also be calculated. Laboratory based analytical validity and reliability outcomes will be descriptively reported and discussed across research-grade, commercial and novel multi-modal wearable devices.

To examine the analytical (concurrent) validation of running gait outcomes measured in real-world settings from the various grades of wearables in all real-world testing participants (n = 40), we will compare outcomes across the wearable devices (research, commercial and novel multi-modal) through widely used measures of agreement. The absolute difference, r, ICC, the level of agreement through the Bland Altman plots, 95% LoA, RMSEs will be calculated to evaluate agreement between outcomes generated by each wearable [48].

*Exploratory clinical validation analysis*. To provide an initial exploration of clinical (performance) validation, we will investigate group difference and relationships with clinically (or performance) relevant measures. For group differences (e.g., previous injury history or performance level), analysis of co-variance (ANCOVA), will be conducted to assess for differences between groups (injured vs non-injured, or elite vs novice) in the individual running gait outcomes (e.g., ground contact time etc.) from the novel multimodal, commercial and research-grade wearable devices during the running tasks, while controlling for demographics that are significantly related to the running gait outcomes. Relationships between running gait outcomes and demographic variables (e.g., sex, age, height, weight etc.) within groups (injured vs non-injured, or elite vs novice) will be completed through Pearson correlations within each grade of wearable device (research, commercial, multimodal).

To examine the usability of the wearable devices the SUS scores for each wearable will be calculated and visualised. To investigate differences in participant perception of the usability for each grade of wearable, separate analysis of variance (ANOVA) tests will be performed. The ANOVAs will determine the differences between the SUS scores within the different device grades between participant groups (e.g., injury history, performance level). The relationships between the SUS scores, demographics (e.g., sex, age, age-grade performance), and wearables characteristics (e.g., price, weight, and battery life) will be explored using Pearson correlations [39]. The strength of the correlation will be assessed based on Cohen’s criteria: correlations < 0.30 are considered small, correlations between 0.30 and 0.50 will be considered medium, and correlations > 0.50 are considered strong [53].

#### Data management and availability

The study complies with the General Data Protection Regulation (GDPR) and Data Protection Act 2018, which require data to be de-identified as soon as it is practical to do so. All data samples to be collected as part of this study will be anonymised with participants being assigned a unique study code. Any information pertaining to personal details will be kept in locked filing cabinets in the biomechanics laboratory Northumbria University and is only available to research staff directly running the study. All data will be entered into an electronic database using unique study codes for each participant and securely stored on a password-protected computer database. Any significant protocol modifications during this study will be communicated to the trial registry. Data will be available from the study, once conducted, upon reasonable request from the principle investigator (SS). Results will be disseminated through academic outputs (e.g., national, and international conferences and publications).

## Discussion

There is a growing need for simple, valid, reliable, and objective methods to evaluate the running gait away from the laboratory within real-world environments, yet with the availability of such a wide range of research-grade, commercial and high-end multimodal wearables users may find it difficult to select the most suitable [21]. Therefore, through evaluating validity and reliability of various grades of wearables and demonstrating their efficacy and usability in applied studies, this protocol seeks to enhance the understanding of wearables for objective measurement of running gait.

### Analytical validation

This pilot study protocol was developed in response to recently reviewed evidence and study recommendations for assessing gait in ecologically valid environments [54]. Prior to applying wearables to sporting contexts it is critical to examine the validity and reliability of such technology. This proposed protocol will assess the validity and reliability of different grades of wearables (research-grade, commercial, novel multimodal) for running gait assessment, examining outcomes against ‘gold-standard’ reference measures (3D motion capture). Research-grade wearable devices have been developed by academic researchers, but few have been used in applied studies to inform clinical or performance decisions [21]. Similarly, despite the widespread use, fewer than 10% of commercially available wearables are validated against an accepted ‘gold-standard’ [27]. Reliability studies of wearables for running gait are less established [21]. An important implication of this study is that it is expected to provide the first evidence on the accuracy and reliability of using a multi-modal wearable device (DANU Sports System) during running gait, which will help develop the standard of wearables. At present, most wearables are limited in their assessments to only examination of selective spatiotemporal or kinematic outcomes, for example step frequency, step length, tibial acceleration and ground contact time are commonly cited [21]. Despite numerous studies establishing that running biomechanics cannot be described based on a single parameter [55–58]. Therefore, the use of a multi-modal system (e.g., combination of IMU and pressure insoles, or accelerometer and pressure insole) as proposed in this protocol will address such limitations and allow for more comprehensive assessment of running gait.

### Exploratory clinical validation

Following the examination of analytical validity and reliability the protocol will seek to examine runners in a more natural running environment to provide initial evidence on clinical (or performance) validity (i.e., do the analytically valid outcomes provide something meaningful). By moving beyond the laboratory to more natural running environments (i.e., indoor, or outdoor running tracks, or sports venues), we will be able to evaluate whether the different grades of wearables (research-grade, commercial, novel multimodal) acceptably measure and provide meaningful insight in the specified context of use [23]. This study will determine what grade of wearable may be necessary to provide meaningful data on real-world running. The use of sub-group analyses will elucidate outcomes that are most representative of an individual’s running gait, as well as reveal the interactions between confounding demographic factors, injury status and performance level aid performance enhancement and reduce injury risk / occurrence [59–61]. The proposed protocol will allow for examination of such factors and hopes to provide clarity for researchers and practitioners on the applicability of certain wearables in context.

### Usability

Technological, design and aesthetical characteristics are key areas together that affect the adoption of wearable devices in sport [62–64]. Wearable devices such as the ones described in this paper are able to monitor the performance of the user in a cost effective, accurate and non-invasive manner, yet they are of little use if they lack practicality, comfort, and aesthetical pleasure. Hence, a necessary step is to investigate the opinions of the targeted end-users. This protocol will seek to for investigate the usability of wearables during running activity via administration of a questionnaire. With any new technology it is essential to understand user acceptance in order to predict behavioural intention to use the system in the future. Therefore, by understanding user acceptance of the three systems we can gain further insight into future usage.

### Practical implications

Understanding running gait is crucial in injury prevention and for improving performance. Creating a protocol that considers the validity and reliability of wearables for running gait assessment, consequently, allows practitioners to examine numerous key areas (i.e., fatigue, speed, surface, injury) in ecologically valid settings and without disrupting an individual’s training regime. Furthermore, the proposed protocol partly addresses some additional limitations observed in previous studies, including small sample sizes, examination of only one outcome and lack of consideration for the user’s comfort. Through using various wearables in both the lab and real-world environments, the suitability of each device not only in regard to running gait outcomes obtained but also practicality, time and cost-effectiveness, and user comfort can be evaluated.

## Conclusion

This exploratory pilot study will provide evidence for the validity and reliability of running gait as measured via wearables of various grades (research-grade, commercial, novel multimodal) in adult runners. In addition, it will provide initial evidence on the relationships between demographic factors, injury status, and performance level on objectively measured running gait outcomes from different grades of wearables.

## Supporting information

S1 Appendix Pre-testing questionnaire. (DOCX)

S1 Checklist SPIRIT-Outcomes 2022 checklist (for combined completion of SPIRIT 2013 and SPIRIT outcomes 2022 items)a. (PDF)

S1 Data (PDF)

S1 File (DOCX)
