# Supplementary figures and images for: Wearables for running gait analysis: A study protocol
Source: PLoS One. 2023 Sep 11;18(9):e0291289. doi: 10.1371/journal.pone.0291289 (PMC10495009; doi:10.1371/journal.pone.0291289)

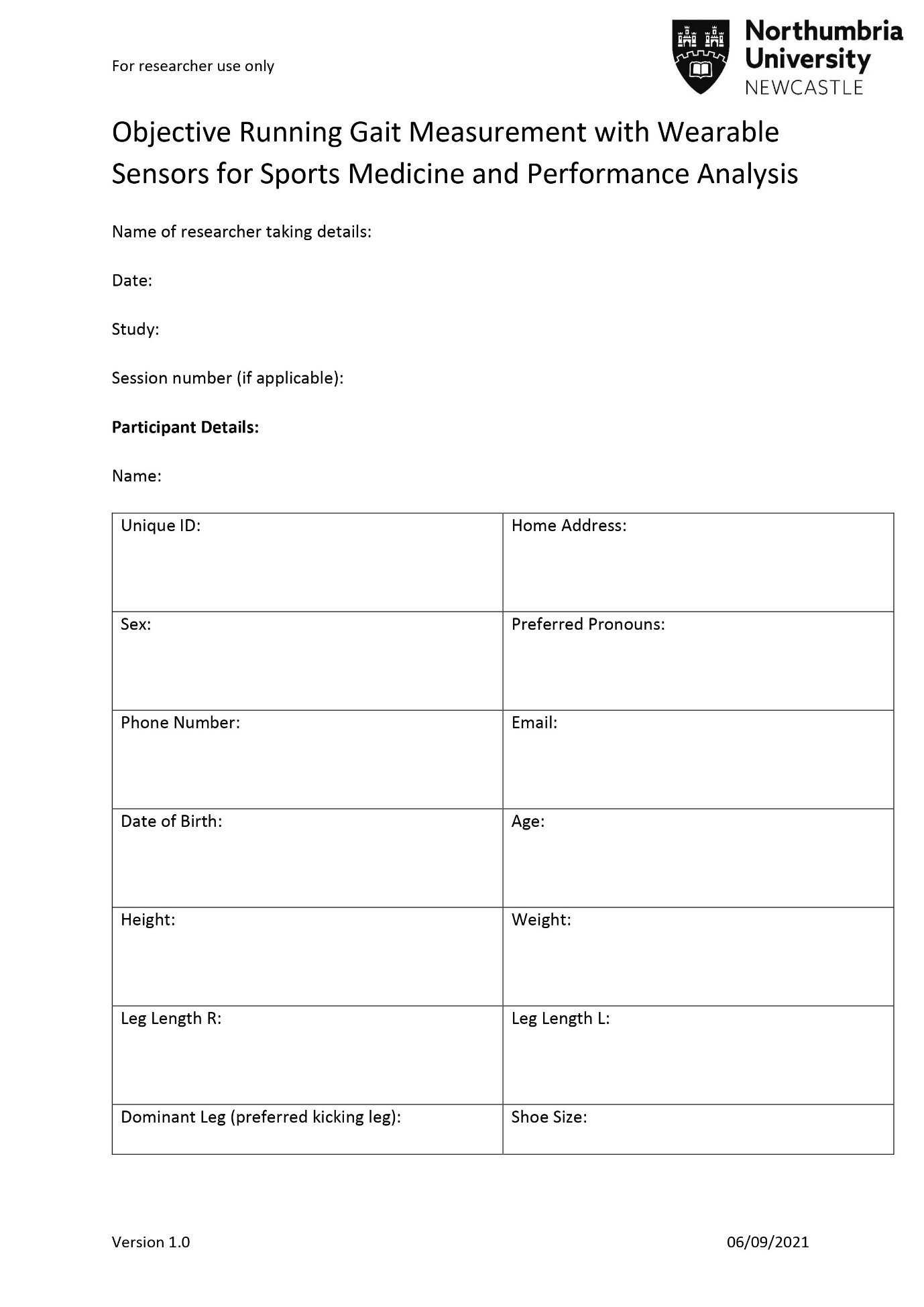
 ***S1 Appendix.*** **Pre-testing Questionnaire.**


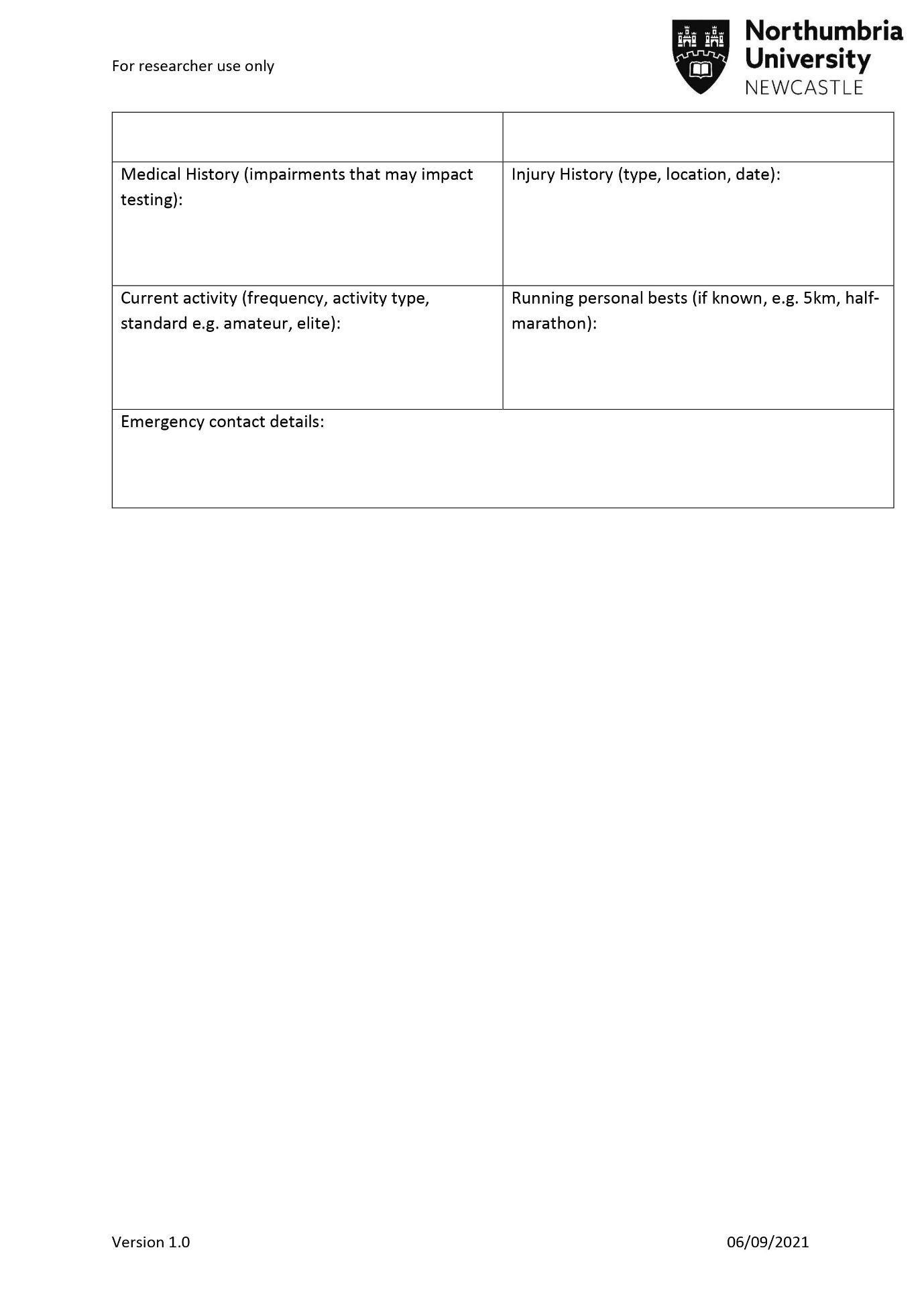

Supplement: S1 Appendix — (DOCX) [file pone.0291289.s001.docx]
