# Supplementary material for: Wearables for running gait analysis: A study protocol
Source: PLoS One. 2023 Sep 11;18(9):e0291289. doi: 10.1371/journal.pone.0291289 (PMC10495009; doi:10.1371/journal.pone.0291289)
Supplement: S1 Data — (PDF) [file pone.0291289.s003.pdf]

My Documents

Amendments

Create New Amendment   Refresh

SUBMISSION ID	CREATED DATE TIME	CREATED BY	STATUS	DESCRIPTION	UPDATED DATE TIME	COORDIN...
No items to display.						

Submission

Submission Ref 33358

Status Approved

Submission Coordinator Claire Thornton

Name rachel.mason rachel.mason

Email

Faculty Health and Life Sciences

Department Sport, Exercise & Rehabilitation: Reh

Submitting As PGR - Postgraduate Research studen

Externally Approved Note: ONLY tick this box if your project has already received full ethical approval from an external organisation

Module Level Approval *Tick this box if staff and this submission refers to an entire module.*

** Only to be used for low or medium risk projects as categorised by the diagnostic risk question set **

Module Code *Type a value* Help

Module Tutor Find Help Clear

Titl...

De...

Em...

Research Supervisor Sam Stuart Find Help Clear

Titl... VC Senior Fellow

De... Health and Life Sciences

Em...

Ethical Risk Level

High

Click here to answer the ethical risk questions

Ethical Risk Diagnostic Questions and Responses

Refresh

ID	QUESTION	ANSWER
1	Gathering data or information from human participants (e.g. via questionnaire / interview/survey/experiment/ social media/ VR)?	YES
2	Collecting personal data, i.e. name, email, home address, computer IP address, phone number etc?	YES
3	Analysis of secondary data NOT in the public domain (e.g. archive material that require organisational membership)?	NO
4	The collection or use of information which is 'commercially sensitive'?	NO
5	Financial inducements other than expenses and compensation for time?	NO
6	Gathering data/information at a physical location external to Northumbria University campuses, franchised locations, and not your normal place of work?	YES
7	Collection of samples such as plants, soils etc, that might disturb the environment or archaeological remains?	NO
8	Research involving animals or materials derived from animals?	NO
9	Anything else which means that the research poses greater than minimal ethical risk?	NO
10	Discussion of highly sensitive topics, including, but not exclusively: bereavement; sexual behaviour, drug use; abuse or exploitation; trauma; pornography; bullying?	NO
11	Potentially vulnerable people or groups, for example children and young people (under 18s), or those who might lack capacity to consent, for example, a learning disability, dementia, or cognitive impairment?	NO
12	Intrusive interventions: the use of drugs or other substances (e.g. food, drink, placebos or drugs); procedures involving physical distress e.g. prolonged or repetitive testing; ionising radiation involving participants which requires external approval through IRAS the process for which is detailed on the ethics and governance web pag emotional distress (e.g. stress or anxiety)?	NO
13	Funding from a source that may be controversial (e.g. due to the nature of the funder, or a conflict of interest)?	NO
14	Covert methods of investigation or deception?	NO
15	International partners, or research undertaken outside of the UK where there may be issues of local practice an political sensitivities? (In these instances it will be necessary to act in accordance with the legal and ethics revie requirements in the countries included in the research and demonstrate awareness of these.)	NO
16	Access to records of personal or sensitive confidential information, including genetic or other biological information concerning identifiable individuals?	NO
17	Individuals or groups where permission of a gatekeeper is normally required for initial or continued access to participants (e.g. NGOs, community leaders)?	NO

18	Recruitment or collection of data from patients, staff or volunteers via the NHS, or social care settings (e.g., home, or residential care)?	NO
19	The collection of bodily tissue e.g. blood, saliva, urine samples from living or deceased persons?	NO
20	A health related study or clinical trial of an investigational medicinal product or a medical device?	NO
21	Direct testing on animals or materials derived from animals?	NO
22	Work that involves direct observation of, or participation in, activities during which it is anticipated that illegal activity, or regulatory breach is likely to occur (e.g. hunting, drug dealing, accessing the dark web, hacking)?	NO
23	Access to or collection of data, information, materials (e.g. magazines, publications, websites, and social media) relating to extremism, radicalisation or terrorism (including extreme or terror groups)?	NO
24	Funding/ sponsorship from, or the involvement of, the UK Ministry of Defence, Military (UK and International), an or, EU Security funding call?	NO
25	The collection of data/information that might be confidential or classified (e.g. protected by the Official Secrets Act)?	NO
26	Other considerations that mean that this research should be treated as 'high risk'?	NO
27	Face to face research with human participants during the Covid-19 pandemic?	**YES**

Co-investigators

Add Edit Delete Save Refresh

NAME OF CO-INVESTIGATORS

Dr Samuel Stuart

Dr Gillian Barry

Dr Alan Godfrey

Oisin Lennon

G1: General Aims and Research Design (Mandatory)

Title

Title of your research project

Objective Running Gait Measurement with Wearable Sensors for Sports Medicine and Performance Analysis

Outline General Aims and Research Objectives

State your research aims/questions (maximum 500 words). This should provide the theoretical context within which the work is placed, and should include an evidence-based background, justification for the research, clearly stated hypotheses (if appropriate) and creative enquiry.

Running gait is impacted by previous injury, or fatigue and can separate athletes at differing performance levels. It requires important clinical components, such as intact musculoskeletal, neurological and sensory systems that can be impacted injury. Similarly, gait patterns may be a useful to observe the efficiency of running across different performance levels to aid in training and development. Having an accessible and accurate method of evaluating running gait would enable use of a range of health and performance outcomes. Motion capture and force plate analysis of running can provide large amounts of high-quality data. However, there is evidence that repeated measurements of over ground laboratory running may result in data that is not representative of running gait, this may be due to numerous factors such as: participants who are aware of an ongoing measurement are likely to modify their running gait; it is not their normal running environment; the distance and number of trials analysed may not be representative and limit the generalisability of results; the large number of markers may also alter technique. Additionally, the expense of laboratory references means that they are not readily available for athletes, clinicians, coaches or others involved in sport. Wearable technology is an alternative approach that has the potential to overcome these limitations, as they are relatively low-cost and can quantify running biomechanical patterns in a natural environment. Furthermore, they are designed to be small, lightweight, wireless and unobtrusive. At present, running studies utilising wearable sensors have primarily focused on determining injury status, examining runners of different experience, determining the effect of fatigue, or detecting gait phase characteristics during running such as heel-strike and toe-off events, stride time or foot strike pattern. A recent development in wearable technology are novel multi-modal devices (combined pressure sensor and inertial measurement unit) that can be used in any environment. This project aims to examine the use of wearable technology to examine running gait, specifically evaluating the validity/reliability and application of the device for sports injury and performance evaluation.

OBJECTIVES
1) To investigate the validity and the reliability of wearable technology during running against gold standard measures.

2) To examine whether running gait measured using the wearable technology can differentiate athletes with and without a history of lower limb injuries.

3) To examine whether running gait objective outcomes obtained from the wearable technology can differentiate between athlete performance level.

G2: Research Activities (Mandatory)

Please give a detailed description of your research activities

Please provide a description of the study design, methodology (e.g. quantitative, qualitative, practice based), the sampling strategy, methods of data collection (e.g. survey, interview, experiment, observation, participatory), and analysis. Do sensitive topics such as trauma, bereavement, drug use, child abuse, pornography, extremism or radicalisation inform the research? If so have these been fully addressed?

The overall project / study will be conducted with two sub-studies; 1) Laboratory Validation and 2) Real-world Environment, which will use very similar methodologies.
Note: This project will involve two studies (Laboratory validation and Real-world Environment), that will involve two cohorts of 40 people (80 people in total). For study aims 1 and 2 will use the same participants that will be involved in the gait laboratory testing. Study aim 3 will involve a separate cohort of participants.
Overall study design: Quantitative, observational. Use of questionnaires (see below and G6).
Sub-Study 1: Laboratory Validation: Investigate the validity and reliability of a novel wearable technology (DANU Sport Socks) in those with and without a history of lower limb injuries.
Locations: Northumbria University gait laboratory
Recruitment strategy: Volunteers who meet the inclusion criteria: (a) be fluent in English, b) participate in running activities of some form (e.g. recreationally, competitively, part of a team sport), c) aged 18 -55 years, d) No history of physical, cognitive impairment that would significantly impact mobility and or ability to follow instructions/tasks).
Participants will be recruited from the UK (e.g. university teams, sporting clubs and the general population, via word of mouth and social media. (see G6 for recruitment flyer/post for social media)). The sample group will consist of 40 adults (aged 18-55 years) participants regardless of their gender, sexuality, race will be involved in the study. The sample will include 20 participants with a history of, 20 participants without lower-limb injury, and an even split of sexes will be used.
Equipment: 3D motion capture (Vicon motion systems) (including video data); Force Plates/Pressure Mats (AMTI force plates); Wearable sensors (DANU socks, Axivity ax6, Opal Sensors); Treadmill
Due to the nature of the study, video data will recorded as part of the 3D motion capture data collection and also for analyses purposes. All data will be anonymised unless participants give consent for their facial features to be recognisable in video or photos. The purpose of this would be for training, publication and conference presentations
3D motion capture markers will be placed according to the Vicon lower limb model (bilaterally on the pelvis, thigh, knee, tibia, ankle, toe, heel).
Wearable Technology: The novel DANU Sports Socks comprise of 15 silicone based capacitive sensors, where the sole of the foot is divided into 3 columns and 5 rows of data. Furthermore, the DANU system contains an IMU pod which when the socks are worn will be located on the distal antero-medial aspect of the tibia. The IMU pod contains a dual tri-axial accelerometer (±16g and ±2g), gyroscope (2000 ± °/s) and magnetometer (± 1.5 Gauss). The DANU Sports Socks will be sampled at 60 Hz and will be wirelessly connected by Bluetooth to a computer running DANU's software to initiate and stop data recordings. Trial data will be saved to the storage chip embedded in the insoles and downloaded afterwards via USB cable. 5 Opal inertial sensors (version 1; APDM Inc, 2000 deg/sec gyroscope, magnetometer and sampling frequency of 128 Hz), will be attached to the fifth lumbar vertebrae (L5) and both wrists and feet (dorsally); held on via Velcro straps. The Axivity (AX6; 100Hz accelerometer and 2000 deg/sec gyroscope) will be secured via double-sided tape, applied directly to the skin (e.g. at lumbar region L5). Each sensor wirelessly synchronized with the laptop after each task.
Protocol: Participants (n=40) will be asked to attend two sessions (1 week apart) held in the Gait Laboratory Northumbria University at a date that is convenient to them. The total time to complete this element of study will be approximately 2 hours. After being briefed and signing a consent form, the investigator will collect participant information (e.g. age, injury status, sport, dominant side, activity levels, shoe size), anthropometric data (e.g. height, weight, leg length).
The researchers will then ask participants to wear a number of sensors (non-invasive) attached either directly to the skin (with hypoallergenic skin adhesives), with a strap or as part of a garment (socks) on pre-determined locations (stated below). Further markers will be placed at pre-determined locations (stated below) to capture 3D motion data. Participants will be asked to complete a battery of sub-maximal mobility / movement tasks (detailed below). All 40 participants will be asked to repeat the testing a approximately week later for reliability purposes, whereby participants would complete the same testing protocol. This would be arranged for a time that suits them.
Testing Protocol (to be completed at baseline and a week later): The mobility / movement assessment will involve 4 different activities, detailed below;
1) Balance assessment (e.g. standing still / static for up to 120 secs) including e.g. single leg stance on foam surface with eyes closed (both legs), single leg stance on firm surface with eyes closed (both legs), feet apart on firm surface with eyes open, feet apart on firm surface with eyes closed, tandem feet on firm surfaces with eyes closed, tandem feet on foam surface with eyes closed.
2) Jumping assessment e.g. counter-movement jump, pogo jump, lateral hop, drop jump, 3 trials per task.
3) Walking assessment e.g. 3 speeds (self-perceived slow, medium fast), 2 surfaces (treadmill and over-ground), 3 trials lasting up to 120 seconds.
4) Running assessment e.g. running on a treadmill or overground. Treadmill running: at 3 speeds (10, 12, 14 km/hr), for 5 minutes at each speed. Over-ground running: self-selected speed, 10 trials running over the force plates (per foot)).
Participants will be allowed adequate time to familiarise themselves with the protocol, as well as take rest / refreshments when needed.
Questionnaires: After the mobility / movement assessments, researchers will ask participants to complete the system usability questionnaire to assess perceptions of usability and comfort of the technologies (this takes approximately 5-10 minutes) (see below and G6).
After participants have completed the study the researcher will give the opportunity to discuss the research further how they can find out about the results, and how they can withdraw their data if they wish (De-brief sheet; see attached in documents section).
Outcome measures: The primary outcome for this study will be Ground Contact Time (the most commonly reported running gait metric from wearable sensors). Secondary running gait measures will include; Running speed, Centre of Mass acceleration, Foot-strike Mechanics/Pattern, Cadence, Stride Velocity, Stride-Length, Peak Power, Peak Acceleration, Jump Height, Flight-Time, Ground Reaction Forces, Symmetry Index, Strike Index, Joint angles, Joint velocities
Data / Statistical analysis: Statistical analyses will be performed in SPSS Statistics 26 (IBM Corp., Armonk, USA). All dependent variables will be checked for normality using the Shapiro–Wilk test.
For validity, we will use the full cohort (n=40) and apply one-way repeated measure ANOVAs to test for differences between the two measurement systems (DANU Sport vs laboratory references). Pearson's product-moment correlation coefficient will be calculated to assess the relative agreement between the systems. Furthermore, Bland-Altman plots will be constructed to assess the inter-instrument agreement between the gold standard reference measures and the DANU system. Mean bias between the systems and 95% limits of agreement (LoA) will also be calculated . Absolute agreement intraclass correlation coefficient (ICC) model will be used to assess reliability. ICCs will be interpreted as follows: ICC < 0.75 poor to moderate, ICC 0.75–0.89 good, and ICC > 0.90 excellent. The standard error of measurement (SEM) and smallest detectable change (SDC) will also be calculated as part of reliability. Cases with missing data will be excluded from analyses on those variables where data was absent.
For group (previous injury vs no previous injury) differences, independent t-tests will be conducted to assess for differences between the outcomes from the wearable sensors during the mobility / movement tasks.
Sample Size Justification: There have been no previous studies that have used the DANU Sport Socks, and we do not have [illegible]

any pilot data to base a specific sample size estimation on. However, our recent extensive literature review (n>100 studies) has shown that previous research that have found statistically significant outcomes when investigating wearable technology for running gait assessment with sample sizes ranging from 1 to 60, with the average number of participants at 10. Thus, we expect that a sample size of 40 participants (20 per group) will provide adequately powered data for our study aims / outcomes, particularly in relation to potentially niche samples required (those with lower limb injuries).
For laboratory validation, we require a minimum of 30 participants to ensure adequate data for parametric analysis (Central Limits Theorem). For the group comparison (previous injury vs no previous injury), based on a previous recent study (DeJong Lempke et al. 2021) that examined wearable sensor outcomes in 32 runners per previous injury group, with a Cohens d effect size of 0.9 for ground contact time differences between groups, we require a minimum of 16 people per group to ensure adequate data (power 0.8, probability level 0.05). Therefore, our sample of n=40 people, with 20 people per previous injury group will allow for adequate data collection, with an additional 20% (n=8) included in case of drop-outs for the reliability element of the study.
Sub-Study 2: Real-World Environment: To examine whether running gait objective outcomes obtained from novel wearable technology (DANU Sport Socks) can differentiate between athlete performance level.
Locations: Local or University Sports Clubs (e.g. Sunderland A.F.C., Northumbria University RFC, Tyne Bridge Harriers, Northumbria University sports clubs)
Recruitment strategy: Volunteers who meet the inclusion criteria: (a) be fluent in English, b) participate in running activities of some form (e.g. recreationally, competitively, part of a team sport), c) aged 18 -55 years, d) No history of physical, cognitive impairment that would significantly impact mobility and or ability to follow instructions/tasks).
Participants will be recruited from researcher connections and word of mouth (see M1). The sample group will consist of 40 adults (aged 18-55 years) participants regardless of their sex, sexuality, race will be involved in the study. The participants will be stratified based on their experience level (i.e. 20 amateur, 20 sub-elite/elite athletes), and an even split of sexes will be used. Prior to study commencement a talk with the coaches, players/athletes will be arranged to discuss involvement in the study.
Equipment: Video data, treadmill, wearable technology. This study will use the same wearable technology as stated above.
Protocol: Researchers will attend the participants training environment at a date that is convenient to them. After being briefed and signing a consent form, the investigator will ask participants to begin with the tasks. The investigator will collect participant information (injury status, sport, dominant side, activity levels, shoe size), anthropometric data (height, weight, leg length). No sensitive topics such as trauma, bereavement, drug use, child abuse, pornography, extremism or radicalisation inform the research, nor will be incorporated.
Researchers will ask participants to wear a number of wearable sensors (non-invasive). These non-invasive sensors will be attached either directly to the skin (with hypoallergenic skin adhesives), with a strap and double-sided tape, worn in an elasticated belt or as part of a garment (stated above) on pre-determined locations (stated above).
The participants will complete a battery of movement / mobility assessments (detailed below), whilst wearing the wearable technologies. We will also ask participants to complete a questionnaire which assess perceptions of usability and comfort of the technologies following their use (approximately 10 minutes) (see below and G6). After participants have completed the study the researcher will give the opportunity to discuss the research further how they can find out about the results, and how they can withdraw their data if they wish. The total time to complete this study will be approximately 2 hours.
Testing Protocol: The movement / mobility protocol will be conducted in their natural environments (i.e. their running or sporting club setting), involving;
1) Balance assessment (e.g. standing still / static for up to 120 secs) including e.g. single leg stance on foam surface with eyes closed (both legs), single leg stance on firm surface with eyes closed (both legs), feet apart on firm surface with eyes open, feet apart on firm surface with eyes closed, tandem feet on firm surfaces with eyes closed, tandem feet on foam surface with eyes closed.
2) Jumping assessment e.g. counter-movement jump, pogo jump, lateral hop, drop jump, 3 trials per task.
3) Walking assessment e.g. 3 speeds (self-perceived slow, medium fast), 2 surfaces (treadmill and over-ground), 3 trials lasting up to 120 seconds.
4) Running assessment e.g. running on a treadmill or overground. Treadmill running: at 3 speeds (10, 12, 14 km/hr), for 5 minutes at each speed. Over-ground running: self-selected speed, 5 x 80m intermittent runs). Participants will be allowed adequate time to familiarise themselves with the protocol, as well as take rest / refreshments when needed.
5) Real-world running assessment: Participants will be asked to complete a running protocol once a week for 5 weeks wearing the DANU socks (including initial testing). They will be asked to run for a period of 30 minutes each week, at an RPE of 6/7 over a 5-week period and 5 x 80m sub-maximal intermittent runs.
Overall participants will be required to participate in this study for 2 hours on the first session, and 1 hour on the following 4 sessions.
Questionnaires: To assess effort during the testing, participants will be asked to convey their Rating of Perceived Exertion (RPE) (RPE 1-10 scale). The System Usability Scale (SUS) will be used to assess the usability of the wearable technology, and participants will be asked to complete this multiple-choice questionnaire post-testing (approximately 5-10 minutes).
Outcome measures: The primary outcome for this study will be Ground Contact Time. Secondary running gait measures will include; Running speed, Centre of Mass acceleration, Foot-strike Mechanics/Pattern, Cadence, Stride Velocity, Stride-Length, Peak Power, Peak Acceleration, Jump Height, Flight-Time, Ground Reaction Forces, Symmetry Index, Strike Index, Joint angles, Joint velocities
Data analysis: Statistical analyses will be performed in SPSS Statistics 26 (IBM Corp., Armonk, USA). All dependent variables will be checked for normality using the Shapiro–Wilk test. For group (previous injury vs no previous injury) differences, independent t-tests will be conducted to assess for differences between the outcomes from the wearable sensors during the mobility / movement tasks.
Sample Size Justification: Similar to the laboratory study justification, there have been no previous studies that have used the DANU Sport Socks, and we do not have any pilot data to base a specific sample size estimation on. Similarly, there have been no previous studies that have examined running gait in amateurs and elite athletes in real-world environments using wearable sensors (for ground contact time). However, our recent extensive literature review (n>100 studies) has shown that previous research that have found statistically significant outcomes when investigating wearable technology for running gait assessment with sample sizes ranging from 1 to 60, with the average number of participants at 10. Thus, we expect that a sample size of 40 participants (20 per group) will provide adequate data for our study aims / outcomes, particularly in relation to potentially niche samples required (elite athletes). However, we have conducted a sample size calculation for the group comparison (amateur/recreational vs sub-elite/elite), based on a previous recent laboratory study of running gait in amateur and elite runners (Rueda et al. 2017), that examined 38 amateur and 8 elite athletes running and showed a Cohens d effect size of 0.95 for ground contact time differences between groups, we require a minimum sample size of 19 people per group to ensure adequately powered data (power 0.8, probability level 0.05). Therefore, our target sample size of 40 people, 20 per group, will ensure adequate data collection sample.

M1: People and/or Personal Data

✓ Tick if your work involves people and/or personal data?

Sample Groups

Provide details of the sample groups that will be involved in the study and include details of their location (whether recruited in the UK or from abroad) and any organisational affiliation. For most research studies, this will cover: the number of sample groups; the size of each sample group; the criteria that will be used to select the sample group(s) (e.g. gender, age, sexuality, health conditions). If the sample will include NHS staff or patients please state this clearly. If this is a pilot study and the composition of the sample has not yet been confirmed, please provide as many details as possible.

Healthy, adults will be recruited and assessed.

Sub-Study 1: Laboratory Validation: Investigate the validity and reliability of a novel wearable technology (DANU Sport Socks) in those with and without a history of lower limb injuries.
Locations: Northumbria University gait laboratory
Recruitment strategy: Volunteers who meet the inclusion criteria: (a) be fluent in English, b) participate in running activities of some form (e.g. recreationally, competitively, part of a team sport), c) aged 18 -55 years, d) No history of physical, cognitive impairment that would significantly impact mobility and or ability to follow instructions/tasks). For this element of the study the additional criteria of history of lower limb injury (that doesn't affect current participation in running activities) or no history of lower limb injury will be discussed when asking for medical history. Participants will be recruited from the UK (e.g. university teams, sporting clubs and the general population, via word of mouth and social media (see G6 for recruitment flyer/post for social media). The sample group will consist of 40 adults (aged 18-55 years) participants regardless of their gender, sexuality, race will be involved in the study. The sample will include 20 participants with a history of, 20 participants without lower-limb injury, and an even split of sexes will be used.
Sample Size Justification: There have been no previous studies that have used the DANU Sport Socks, and we do not have any pilot data to base a specific sample size estimation on. However, our recent extensive literature review (n>100 studies) has shown that previous research that have found statistically significant outcomes when investigating wearable technology for running gait assessment with sample sizes ranging from 1 to 60, with the average number of participants at 10. Thus, we expect that a sample size of 40 participants (20 per group) will provide adequately powered data for our study aims / outcomes, particularly in relation to potentially niche samples required (those with lower limb injuries).
For laboratory validation, we require a minimum of 30 participants to ensure adequate data for parametric analysis (Central Limits Theorem). For the group comparison (previous injury vs no previous injury), based on a previous recent study (DeJong Lempke et al. 2021) that examined wearable sensor outcomes in 32 runners per previous injury group, with a Cohens d effect size of 0.9 for ground contact time differences between groups, we require a minimum of 16 people per group to ensure adequate data (power 0.8, probability level 0.05). Therefore, our sample of n=40 people, with 20 people per previous injury group will allow for adequate data collection, with an additional 20% (n=8) included in case of drop-outs for the reliability element of the study.

Sub-Study 2: Real-World Environment: To examine whether running gait objective outcomes obtained from novel wearable technology (DANU Sport Socks) can differentiate between athlete performance level.
Locations: Local or University Sports Clubs (e.g. Sunderland A.F.C., Northumbria University RFC, Tyne Bridge Harriers, Northumbria University sports clubs)
Recruitment strategy: Volunteers who meet the inclusion criteria: (a) be fluent in English, b) participate in running activities of some form (e.g. recreationally, competitively, part of a team sport), c) aged 18 -55 years, d) No history of physical, cognitive impairment that would significantly impact mobility and or ability to follow instructions/tasks).
Participants will be recruited from researcher connections and word of mouth (see M1). The sample group will consist of 40 adults (aged 18-55 years) participants regardless of their sex, sexuality, race will be involved in the study. The participants will be stratified based on their experience level (i.e. 20 amateur, 20 sub-elite/elite athletes), and an even split of sexes will be used. Prior to study commencement a talk with the coaches, players/athletes will be arranged to discuss involvement in the study.
Recruitment of participants will take place through contacts of the researchers involved. Including Dr Glen Rae (Director Medicine and Performance, Durham CCC team), Peter Brand (Head physio, SAFC), Sophie Marr (Head physio, Tyne Bridge Harriers) (see G6 for supporting documents).
(see G6 for supporting documents).
Sample Size Justification: Similar to the laboratory study justification, there have been no previous studies that have used the DANU Sport Socks, and we do not have any pilot data to base a specific sample size estimation on. Similarly, there have been no previous studies that have examined running gait in amateurs and elite athletes in real-world environments using wearable sensors (for ground contact time). However, our recent extensive literature review (n>100 studies) has shown that previous research that have found statistically significant outcomes when investigating wearable technology for running gait assessment with sample sizes ranging from 1 to 60, with the average number of participants at 10. Thus, we expect that a sample size of 40 participants (20 per group) will provide adequate data for our study aims / outcomes, particularly in relation to potentially niche samples required (elite athletes). However, we have conducted a sample size calculation for the group comparison (amateur/recreational vs sub-elite/elite), based on a previous recent laboratory study of running gait in amateur and elite runners (Rueda et al. 2017), that examined 38 amateur and 8 elite athletes running and showed a Cohens d effect size of 0.95 for ground contact time differences between groups, we require a minimum sample size of 19 people per group to ensure adequately powered data (power 0.8, probability level 0.05).
Therefore, our target sample size of 40 people, 20 per group, will ensure adequate data collection sample.

General comments:

Due to the nature of the study, video data will recorded as part of the 3D motion capture data collection and also for analyses purposes. All data will be anonymised unless participants give consent for their facial features to be recognisable in video or photos. The purpose of this would be for data checking (e.g. checking the videos if abnormalities are seen in the wearable sensor data), as well as staff protocol training, publications and conference presentations that will show the protocol being performed

Nature of data pertaining to Living Individuals

If you will be including personal data of living individuals, including still or moving images, please specify the nature of this data, and (if appropriate) include details of the relevant individuals who have provided permission to utilise this data, upload evidence of these permissions in the supporting documentation section.

Details of any Special Category Data - If you will be collecting data revealing racial or ethnic origin, political opinions, religious or philosophical beliefs, trade union membership, data concerning health or data concerning a natural person's sex life or sexual orientation, please specify which categories you will be using.

Data will be collected using 3D motion capture, video and wearable sensors to monitor how participants perform a battery of tests (see in G2).
Nature of data: Quantitative numerical data (e.g. motion capture and wearable sensors). Outcome measures include: Running speed, Centre of Mass acceleration, Ground Contact Time, Foot-strike Mechanics/Pattern, Cadence, Stride Velocity, Stride-Length, Peak Power, Peak Acceleration, Jump Height, Flight-Time, Ground Reaction Forces, Symmetry Index, Strike Index, Joint angles, Joint velocities. Additional outcomes from SUS questionnaires regarding usability of the devices (see G6)

Personal data relating to name, age, height and general health status will be collected only with the participant information sheet. No information relating to finance, sexual orientation, religion or political views will be collected and stored.
All data will be anonymised unless participants give consent for their facial features to be recognisable in video or photos. The purpose of this would be for data checking (e.g. checking the videos if abnormalities are seen in the wearable sensor data), as well as staff protocol training, publications and conference presentations that will show the [illegible]

Legal Basis for Processing: Further guidance can be found here

If you require further information, please contact the Data Protection Officer by emailing

dp.officer@northumbria.ac.uk

GDPR Article 6(1) (e).... “processing is necessary for the performance of a task carried out in the public interest”.

Recruitment

Describe the step by step process of how you will contact and recruit your research sample and name any organisations or groups that will be approached. Your recruitment strategy must be appropriate to the research study and the sensitivity of the subject area. You must have received written permission from any organisations or groups before you begin recruiting participants. Copies of draft requests for organisational consent must be included in the ‘Supporting Documentary Evidence’. You must also provide copies of any recruitment emails/posters that will be used in your study.

Sub-Study 1: Laboratory Validation: Investigate the validity and reliability of a novel wearable technology (DANU Sport Socks) in those with and without a history of lower limb injuries.
Recruitment strategy: Volunteers who meet the inclusion criteria: (a) be fluent in English, b) participate in running activities of some form (e.g. recreationally, competitively, part of a team sport), c) aged 18 -55 years, d) No history of physical, cognitive impairment that would significantly impact mobility and or ability to follow instructions/tasks). For this element of the study the additional criteria of history of lower limb injury (that doesn't affect current participation in running activities) or no history of lower limb injury will be discussed when asking for medical history. Participants will be recruited from the UK (e.g. university teams, sporting clubs and the general population, via word of mouth and social media (see G6 for recruitment flyer/post for social media). The sample group will consist of 40 adults (aged 18-55 years) participants regardless of their gender, sexuality, race will be involved in the study. The sample will include 20 participants with a history of, 20 participants without lower-limb injury, and an even split of sexes will be used.

Sub-Study 2: Real-World Environment: To examine whether running gait objective outcomes obtained from novel wearable technology (DANU Sport Socks) can differentiate between athlete performance level.
Recruitment strategy: Volunteers who meet the inclusion criteria: (a) be fluent in English, b) participate in running activities of some form (e.g. recreationally, competitively, part of a team sport), c) aged 18 -55 years, d) No history of physical, cognitive impairment that would significantly impact mobility and or ability to follow instructions/tasks).
Participants will be recruited from researcher connections and word of mouth (see M1). The sample group will consist of 40 adults (aged 18-55 years) participants regardless of their sex, sexuality, race will be involved in the study. The participants will be stratified based on their experience level (i.e. 20 amateur, 20 sub-elite/elite athletes), and an even split of sexes will be used. Prior to study commencement a talk with the coaches, players/athletes will be arranged to discuss involvement in the study.
Recruitment of participants will take place through contacts of the researchers involved. Including Dr Glen Rae (Director Medicine and Performance, Durham CCC team), Peter Brand (Head physio, SAFC), Sophie Marr (Head physio, Tyne Bridge Harriers) (see G6 for supporting documents).

Informed ethical consent will be obtained from all participants prior to commencing the assessment procedures. Consent will be recorded with a paper consent form. Information sheet, debrief sheet and consent forms are provided in additional files (G6).
Participants will only be identifiable if they provide consent for video or photos where their facial features will be recognisable. The purpose of this would be for data checking (e.g. checking the videos if abnormalities are seen in the wearable sensor data), as well as staff protocol training, publications and conference presentations that will show the protocol being performed

☐ **Remuneration**

Details of remuneration

Will you make any payment or remuneration to participants or their carers/consultees? If yes: Please provide details/justifications. Note that your Faculty may have specific guidelines on participant payments/payment rates etc and you should consult these where appropriate.

No remuneration to participants will be made.

Type of Consent

Informed Consent

Type of Consent Details

Please include copies of information sheets and consent forms in the ‘G6: File Attachments' section. If the study involves participants who lack capacity to consent, procedures in line with sections 30-33 of the Mental Capacity Act will need to be put in place. If you are using alternative

formats to provide information and /or record consent (e.g. images, video or audio recording), provide brief details and outline the justification for this approach and the uses to which it will be put:

Informed Consent attached in G6
Participant Information Sheet attached in G6

Researcher and Participant Safety Issues

If there any risks the research could cause any discomfort or distress to participants (physical, psychological or emotional) describe the measures that will be put in place to alleviate or minimise them. Please give detailsof the support that will be available for any participants who become distressed during their involvement with the research.

Minimal and low risks of distress or discomfort to participants.
Minimal and low risk from taking apart, all of the tasks participants will be recruited based on tasks they are familiar with, therefore resemble their normal daily lives and sporting activities (e.g. walking, running, balance, jumps) and the non-invasive wearable technologies won't interfere with any of their ability to do these, or cause any discomfort. Participants may possibly experience muscular fatigue during and in the days following completing the protocol. Additionally there is a very small chance of minor injury such as would be associated with sub-maximal running (e.g. muscle strain).

All wearable devices will be cleaned and sterilised using medical grade Clinell Universal cleaning wipes wipes before and after use.
All equipment will be serviced in line with manufacturers recommendations and that it has a valid PAT test date (where appropriate). If it does not, the equipment will not be used and they will inform the Technical team who will arrange PAT testing.
Participant to be instructed how to take off and reattach the device for general hygiene purposes.

Risk assessments can be found in G6

Additional comments:
Laboratory equipment includes wearable technologies, 3D motion capture system, force plates and an instrumented mat/carpet. All sessions will be supervised and data will be stored on University OneDrive as well as a password-protected hard drive accessible by the researchers only. Volunteers will be asked to wear numerous lightweight and low-cost inertial measurement units (wearable) which attach directly to the body. Some attach directly to the skin with hypoallergenic skin adhesives and covered with an additional Hypafix bandage for extra support. All wearable technologies are battery-powered and safe to wear for extended periods of time on the body/skin.
Wearable technology records and logs inertial data only which is tagged to the participant by a unique identifier number, i.e. all data is anonymous.

All data will be anonymised unless participants give consent for their facial features to be recognisable in video or photos. The purpose of this would be for data checking (e.g. checking the videos if abnormalities are seen in the wearable sensor data), as well as staff protocol training, publications and conference presentations that will show the protocol being performed

Data Gathering Materials Used

Provide a detailed description of what the participants will be asked to do for the research study, including details about the process of data collection (e.g. completing how many interviews / assessments, when, for how long, with whom). Add any relevant documentation to the 'Supporting Documentary Evidence' section of this form.

Sub-Study 1: Laboratory Validation: Investigate the validity and reliability of a novel wearable technology (DANU Sport Socks) in those with and without a history of lower limb injuries.
Locations: Northumbria University gait laboratory
Equipment: 3D motion capture (Vicon motion systems) (including video data); Force Plates/Pressure Mats (AMTI force plates); Wearable sensors (DANU socks, Axivity ax6, Opal Sensors); Treadmill
Due to the nature of the study, video data will recorded as part of the 3D motion capture data collection and also for analyses purposes. All data will be anonymised unless participants give consent for their facial features to be recognisable in video or photos. The purpose of this would be for training, publication and conference presentations
3D motion capture markers will be placed according to the Vicon lower limb model (bilaterally on the pelvis, thigh, knee, tibia, ankle, toe, heel).
Wearable Technology: The novel DANU Sports Socks comprise of 15 silicone based capacitive sensors, where the sole of the foot is divided into 3 columns and 5 rows of data. Furthermore, the DANU system contains an IMU pod which when the socks are worn will be located on the distal antero-medial aspect of the tibia. The IMU pod contains a dual tri-axial accelerometer (±16g and ±2g), gyroscope (2000 ± °/s) and magnetometer (± 1.5 Gauss). The DANU Sports Socks will be sampled at 60 Hz and will be wirelessly connected by Bluetooth to a computer running DANU's software to initiate and stop data recordings. Trial data will be saved to the storage chip embedded in the insoles and downloaded afterwards via USB cable. 5 Opal inertial sensors (version 1; APDM Inc, 2000 deg/sec gyroscope, magnetometer and sampling frequency of 128 Hz), will be attached to the fifth lumbar vertebrae (L5) and both wrists and feet (dorsally); held on via Velcro straps. The Axivity (AX6; 100Hz accelerometer and 2000 deg/sec gyroscope) will be secured via double-sided tape, applied directly to the skin (e.g. at lumbar region L5). Each sensor wirelessly synchronized with the laptop after each task.
Protocol: Participants (n=40) will be asked to attend two sessions (1 week apart) held in the Gait Laboratory Northumbria University at a date that is convenient to them. The total time to complete this element of study will be approximately 2 hours. After being briefed and signing a consent form, the investigator will collect participant information (e.g. age, injury status, sport, dominant side, activity levels, shoe size), anthropometric data (e.g. height, weight, leg length).
The researchers will then ask participants to wear a number of sensors (non-invasive) attached either directly to the skin (with hypoallergenic skin adhesives), with a strap or as part of a garment (socks) on pre-determined locations (stated below). Further markers will be placed at pre-determined locations (stated below) to capture 3D motion data. Participants will be asked to complete a battery of sub-maximal mobility / movement tasks (detailed below). All 40 participants will be asked to repeat the testing a approximately week later for reliability purposes, whereby participants would complete the same testing protocol. This would be arranged for a time that suits them.
Testing Protocol (to be completed at baseline and a week later): The mobility / movement assessment will involve 4 different activities, detailed below;
1) Balance assessment (e.g. standing still / static for up to 120 secs) including e.g. single leg stance on foam surface with eyes closed (both legs), single leg stance on firm surface with eyes closed (both legs), feet apart on firm surface with eyes open, feet apart on firm surface with eyes closed, tandem feet on firm surfaces with eyes closed, tandem feet on foam surface with eyes closed.
2) Jumping assessment e.g. counter-movement jump, pogo jump, lateral hop, drop jump, 3 trials per task.
3) Walking assessment e.g. 3 speeds (self-perceived slow, medium fast), 2 surfaces (treadmill and over-ground), 3 trials lasting up to 120 seconds.
4) Running assessment e.g. running on a treadmill or overground. Treadmill running: at 3 speeds (10, 12, 14 km/hr), for 5 minutes at each speed. Over-ground running: self-selected speed, 10 trials running over the force plates (per foot)).
Participants will be allowed adequate time to familiarise themselves with the protocol, as well as take rest / refreshments when needed.
Overall participants will be required to participate in this study for 2 sessions each lasting approximately 2 hours, therefore approximately 4 hours in total.

Sub-Study 2: Real-World Environment: To examine whether running gait objective outcomes obtained from novel wearable technology (DANU Sport Socks) can differentiate between athlete performance level.
Locations: Local or University Sports Clubs (e.g. Sunderland A.F.C., Northumbria University RFC, Tyne Bridge Harriers, Northumbria University sports clubs)
Equipment: Video data, treadmill, wearable technology. This study will use the same wearable technology as stated above.
Protocol: Researchers will attend the participants training environment at a date that is convenient to them. After being briefed and signing a consent form, the investigator will ask participants to begin with the tasks. The investigator will collect participant information (injury status, sport, dominant side, activity levels, shoe size), anthropometric data (height, weight, leg length). No sensitive topics such as trauma, bereavement, drug use, child abuse, pornography, extremism or radicalisation inform the research, nor will be incorporated.
Researchers will ask participants to wear a number of wearable sensors (non-invasive). These non-invasive sensors will be attached either directly to the skin (with hypoallergenic skin adhesives), with a strap and double-sided tape, worn in an elasticated belt or as part of a garment (stated above) on pre-determined locations (stated above).
The participants will complete a battery of movement / mobility assessments (detailed below), whilst wearing the wearable technologies. We will also ask participants to complete a questionnaire which assess perceptions of usability and comfort of the technologies following their use (approximately 10 minutes) (see below and G6).
Testing Protocol: The movement / mobility protocol will be conducted in their natural environments (i.e. their running or sporting club setting), involving;
1) Balance assessment (e.g. standing still / static for up to 120 secs) including e.g. single leg stance on foam surface with eyes closed (both legs), single leg stance on firm surface with eyes closed (both legs), feet apart on firm surface with eyes open, feet apart on firm surface with eyes closed, tandem feet on firm surfaces with eyes closed, tandem feet on foam surface with eyes closed.
2) Jumping assessment e.g. counter-movement jump, pogo jump, lateral hop, drop jump, 3 trials per task.
3) Walking assessment e.g. 3 speeds (self-perceived slow, medium fast), 2 surfaces (treadmill and over-ground), 3 trials lasting up to 120 seconds.
4) Running assessment e.g. running on a treadmill or overground. Treadmill running: at 3 speeds (10, 12, 14 km/hr), for 5 minutes at each speed. Over-ground running: self-selected speed, 5 x 80m intermittent runs). Participants will be allowed adequate time to familiarise themselves with the protocol, as well as take rest / refreshments when needed.
5) Real-world running assessment: Participants will be asked to complete a running protocol once a week for 5 weeks wearing the DANU socks (including the initial testing). They will be asked to run for a period of 30 minutes each week, at an RPE of 6/7 over a 5-week period and 5 x 80m sub-maximal intermittent runs.
Overall participants will be required to participate in this study for 2 hours on the first session, and 1 hour on the following 4 sessions. Therefore participants will complete approximately 6 hours in total.

General information that applies across all sub-studies:

After participants have completed the study the researcher will give the opportunity to discuss the research further how they can find out about the results, and how they can withdraw their data if they wish. The total time to complete this study will range according to which participant group they are in and if they require additional rest/breaks, however should last approximately 2 hours. Dependent on the participant group, they may be asked to repeat the testing for reliability purposes, whereby participants would complete the same testing protocol. This would be arranged for a time that suits them.

General comments that apply to both sub-studies:
Outcome measures: Running speed, Centre of Mass acceleration, Ground Contact Time, Foot-strike Mechanics/Pattern, Cadence, Stride Velocity, Stride-Length, Peak Power, Peak Acceleration, Jump Height, Flight-Time, Ground Reaction Forces, Symmetry Index, Strike Index, Joint angles, Joint velocities

Questionnaires: To assess effort during the testing, participants will be asked to convey their Rating of Perceived Exertion (RPE) (RPE 1-10 scale). The System Usability Scale (SUS) will be used to assess the usability of the wearable technology, and participants will be asked to complete this multiple-choice questionnaire post-testing (10 minutes).
Participants will be tested by at least one of the following people: Rachel Mason, Dr Samuel Stuart, Dr Gillian Barry, Dr Alan Godfrey

All data will be anonymised unless participants give consent for their facial features to be recognisable in video or photos. The purpose of this would be for data checking (e.g. checking the videos if abnormalities are seen in the wearable sensor data), as well as staff protocol training, publications and conference presentations that will show the protocol being performed

Potential Ethical Issues

Please describe any potential ethical issues the project may have which are not covered above, and how you have sought to minimise these.

Minimal and low risk from taking apart, all of the task's participants will be asked to do resemble their normal daily lives and sporting activities (e.g walking, running, balance and jumps) and the technologies won't interfere with any of their ability to do these. Participants may possibly experience muscular fatigue during and in the days following completing the protocol. Additionally there is a very small chance of minor injury such as would be associated with sub-maximal running (e.g. muscle strain). Participants will be allowed adequate time to familiarise themselves with the protocol, as well as take rest / refreshments when needed and terminate the testing if they wish.
This study is also observational and will not be used in the decision making and or diagnosis of any medical condition.
All data will be analysed by the research team only and will comply with GDPR regulations
All data will be anonymised unless participants give consent for their facial features to be recognisable in video or photos. The purpose of this would be for data checking (e.g. checking the videos if abnormalities are seen in the wearable sensor data), as well as staff protocol training, publications and conference presentations that will show the protocol being performed

H1: Children (i.e. under 18s) and Vulnerable Adults

☐ Tick if your work involves children or vulnerable adults?

M2: DBS Clearances Required

Add Edit Delete Save Refresh

Do not upload your DBS certificate to this system as this would be contravening General Data Protection Regulations.

Further information relating to DBS Clearance can be found in the Ethics and Governance Handbook using the link below

Ethics and Governance Handbook

******* All fields below relating to DBS certificates must be completed *******

NAME OF PERSON ON CERTIFICATE	TYPE OF DBS CLEARANCE	CERTIFICATE REFERENCE	ADULTS/CHILDREN	DATE OF DBS CERTIFICATE
Rachel Victoria Mason	Basic	001019381697	Adults only	09/06/2021

(Add new row)

M3: Secondary Data

☐ Tick if you will be using secondary data NOT in the public domain?

M4: Commercial Data

☐ Tick if your work involves commercially sensitive data?

M5: Environmental Data

☐ Tick if your work involves the collection of environmental data?

H2: Human Tissue

☐ Tick if your work involves use of human tissue?

H3: Animal Subjects

☐

Tick if your work involves the use of animal subjects (excluding abattoir derived material and commercially sourced fur)?

H4: Security Sensitive Information (including Prevent-related material)

☐ Do you require access to material that is prohibited/restricted (e.g. under Government security classifications or the Official Secrets Act)?

H5: Cultural Sensitivity

☐ Tick if your work involves Art or Artefacts (including culturally sensitive items?)

H6: Reason for resuming face to face research with human participants during Covid-19

Please outline the justification for commencing the research, balancing the risk with the potential benefits of the research, and with reference to the principles that underpin considerations around re-starting research?

• Research should only restart/ start when safe to do so. The safety of research participants and personnel is of paramount importance. If it is possible to undertake the research remotely, this is the preferred option.

• The research must be able to be conducted in a way that aligns with local government guidance as a

minimum. The robustness of these requirements in promoting safeguarding should also be considered and exceeded if needed.

• The research will not require that the researcher or participants puts themselves at any greater risk than they would otherwise pertain as they conduct their daily lives within guidelines set out by the government at the time.

The PhD is a collaborative project between Northumbria University and DANU Sports, therefore in order to meet the needs of the company the research needs to commence. Furthermore, the research involves healthy adult volunteers and participants will be required to follow university policies regarding Covid-19, read participant information sheets and sign informed consent regarding Covid-19 (see G6). If participants are not able or are unwilling to comply to the policies regarding covid-19 they will not be invited to participate in the study. Participants will be asked and reassured about any concerns regarding COVID-19 to ensure that they feel safe and confident to participate.
Regarding safety, all research will be conducted in a manner that follows the latest government and university guidelines. Risk of exposure to COVID-19 for patients and staff has been mitigated, physical access will comply with government restrictions on social distancing, assessment of COVID-19 testing and PPE requirements completed, study arrangements comply with Northumbria university building access policies in respect of COVID-19.
A range of measures to ensure the safety of participants and researchers will be put into place (e.g. increased use of hand sanitizer, social distancing etc.) Risk assessments are provided in G6, where elements regarding Covid-19 and the protocols put into place to minimise the risks are described. The research will not require that the researchers or participants puts themselves at any greater risk than they would otherwise pertain as they conduct their daily lives within guidelines set out by the government at the time.

H7: Research Viability and Capacity during Covid-19

Is this research project feasible from a scientific, financial, or practical point of view during the Covid-19 pandemic?

• For sponsored studies within NHS/ Social care there is an expectation that Sponsors, and sites will work together to conduct a preliminary assessment of all paused studies.

• Delivery of research might be dependent on collaborator, their local arrangements and policies and capacity to undertake the study within the necessary timescales. e.g., health and care services affected by COVID-19 in different ways. It is important for researchers to understand and agree readiness of collaborators and sites where this is essential to the delivery of the research activity.

• Delivery of research must be possible considering the study's population size, proposed location and its cap...

After discussion with the collaborators it has been agreed that it is feasible from all aspects to start the research as regulatory approvals are in place, amendments to time-scales have been agreed, all necessary research funding is confirmed and all relevant bodies are satisfied with the arrangements for public involvement in the study.
Capacity and site readiness; the principal investigator confirmed, as well as additional researchers and collaborators are in place, research management and support in place, all necessary supporting departments (e.g. biomechanics lab) have resource and capacity, all necessary supplies have been procured and are in place (e.g. wearable technology and PPE), study protocol has been checked for integrity to ensure that proposed protocol remains robust and fit for purpose, physical access arrangements for participants have been assessed and are satisfactory (e.g. staggered participant slots, lab capacity numbers)
Risk assessments regarding Covid-19 and the protocols that will be put into place are provided in G6.

G3: Research Data Management Plan (Mandatory)

Anonymising Data (mandatory)

Describe the arrangements for anonymising data and if not appropriate explain why this is and how it is

covered in the informed consent obtained.

All participants will be given a unique alphanumeric ID which will be assigned to them throughout the while study to ensure anonymity. Due to the nature of the study, video data will recorded as part of the 3D motion capture data collection and also for analyses purposes.All data will be anonymised unless participants give consent for their facial features to be recognisable in video or photos. The purpose of this would be for data checking (e.g. checking the videos if abnormalities are seen in the wearable sensor data), as well as staff protocol training, publications and conference presentations that will show the protocol being performed (see G6 for consent forms). Personal data relating to name, age, height and general health status will be collected only. No information relating to finance, sexual orientation, religion or political views will be collected and stored.

Steps that will be followed to anonymise data (i.e. answering 'no' to all):
- Find and highlight direct identifiers
- Visually scan variables
- Can the identity of a participant be known from information in the data file

Due to the nature of the study, video data will recorded as part of the 3D motion capture data collection and also for analyses purposes.All data will be anonymised unless participants give consent for their facial features to be recognisable in video or photos. The purpose of this would be for data checking (e.g. checking the videos if abnormalities are seen in the wearable sensor data), as well as staff protocol training, publications and conference presentations that will show the protocol being performed (see G6 for consent forms)

Storage Details (mandatory)

Describe the arrangements for the secure transport and storage of data collected and used during the study. You should explain what kind of storage you intend to use, e.g. cloud-based, portable hard drive, USB stick, and the protocols in place to keep the data secure.

If you have identified the requirement to collect 'Special category data', please specify any additional security arrangements you will use to keep this data secure.

All paper data (consent form) will be kept in locked storage. All electronic data; including the recordings from your interview, will be stored on the University OneDrive, which is password protected. All data will be stored in accordance with University guidelines and the Data Protection Act (2018). All personal data (name, address, contact details) will be e stored in line with the Data Protection Act and will be destroyed 12 months following the conclusion of the study. Any additional information and data gathered during this research will be stored in line with the Data Protection Act and will be destroyed 72 months following the conclusion of the study. If the research is published in a scientific journal it may be kept for longer before being destroyed. During that time the data may be used by members of the research team only for purposes appropriate to the research question, but at no point will your personal information or data be revealed. Insurance companies and employers will not be given any individual's personal information, nor any data provided by them, and nor will we allow access to the police, security services, social services, relatives or lawyers, unless forced to do so by the courts.

Retention and Disposal (mandatory)

☑ **I confirm that I will comply with the University's data retention schedule and guidance.**

Research Data Management link

General Data Protection Regulations including Data Protection link

Records Retention Schedule link

G4: Research Project Timescale (Mandatory)

Proposed Start Date	04/10/2021
Proposed End Date	31/08/2023

G5: Additional Information

☑ Externally Funded

External Funder: Industrial Funder

Please give details of your 'other' funder

Agresso Reference

IO 120162 (CC 101052)

☐ Franchise Programme Organisation

Please give details of your franchise organisation

Type a value

☐ NHS Involvement

Please give details of any NHS involvement

Type a value

☐ Clinical Trial(s)

Please give details of any Clinical Trial(s)

Type a value

☐ Medicinal Products

Please give details of any Medicinal Product(s)

G6: File Attachments

Additional files can be uploaded e.g. consent documentation, participant information sheet, etc.

Please note: It is best practice to combine all documents into one PDF (This avoids the reviewer having to op...

Go To Attachments

G7: Health and Safety (Mandatory)

☑ I confirm that I have read and understood the University's Health and Safety Policy.

☑ I confirm that I have read and understood the University's requirements for the mandatory completion of risk assessments in advance of any activity involving potential physical risk.

The University Health and Safety Policy can be accessed here

The University Risk Assessment Code of Practice can be accessed here

Please confirm either:

☑ There are PHYSICAL risks associated with the research project work and I confirm that a risk assessment has been approved and attached to this ethics submission.

OR

☐ I can confirm that there are no physical risks associated with this project and so no risk assessments are required.

Students requiring assistance with completing their risk assessment should get in touch with their supervisor or module tutor as the first point of contact. If further assistance is needed, the Faculty Technician can provide further guidance.

For more specific risk assessments (e.g. lab work), especially where the project is Medium or High risk, you are required to consult the Faculty Technical Manager; your Supervisor/Module Tutor will be able to put you in touch.

If you have any questions or concerns, please contact the University Health and Safety Team by emailing

CRHealthandSafety@northumbria.ac.uk

G8: Insurance (Mandatory)

☑ I have read and understood the University Insurance guidance document (link below):

Insurance Guidance link

If you think your activity may involve a High Risk rating or are unsure how to answer the statements - contact fi.insurance@northumbria.ac.uk with a copy of your research proposal for advice.

I confirm my work is covered by University Insurance. I confirm an insurance risk level of:

Medium

If your insurance risk level is HIGH please attach details of exceptional insurance coverage:

Click here to attach a file

G9: Electronic Signature (Mandatory)

- [x] I confirm my supervisor has reviewed the contents of this document
- [x] I confirm I have assessed the ethical risk level of my work correctly and answered the above sections as fully and accurately as possible.

Full Name: rachel.mason

Date: 07 June 2021 15:52:53

PDF Version

Create PDF

No items to display.

Review Comments, Conditions and Outcomes

Log of any Ethical Incidents

Log New Incident

INCIDENT...	CREATED DATE TIME	CREATOR NAME	COMPLAINANT DETAILS

No items to display.

Title and Objectives (see G1)

Add Save

Reviewer A: Approve

Reviewer B: Approve

e.g. Are the research question and/or study aims clear?

DATE	ROLE	COMMENT
7/28/2021	Reviewer A	The objective is that the "project will use novel wearable technology to objectively measure running gait, leading to individualised approaches to sports injury and performance assessment." The submitted project may eventually lead to individual approaches to performance assessment and sports injury down to the road but that is a step beyond what was submitted.
8/10/2021	Reviewer B	There seems to0 be 7 different objectives for the one submission, this seems alot to be committed too and cover.

		It would be best to just focus on 2 or max 3 to start with to establish the need for the further investigations
8/20/2021	PI	We thank the reviewer for their time and effort in providing such detailed and comprehensive feedback for this universit ethics application.<br><br>The specific objectives have been rephrased as follows:<br><br>1) To investigate the validity and the reliability of wearable technology during running against gold standard measures.<br><br>2) To examine whether running gait measured using the wearable technology can differentiate athletes with and without a history of lower limb injuries.<br><br>3) To examine whether running gait objective outcomes obtained from the wearable technology can differentiate between athlete performance level.
9/7/2021	Reviewer B	the new objectives are clearer, more focused and achievable

Proposed Methodology and Analysis (see G2)

Add Save

Reviewer A: Approve

Reviewer B: Approve

e.g. Is the design appropriate to the research question?
Are the methods of data analysis appropriate to the research question?

DATE	ROLE	COMMENT
7/28/2021	Reviewer A	The description is not detailed enough to fully assess the project. When resubmitting provide a detailed description of the research activities.<br><br>- Sample size not provided or justified.<br>- What determines if participants will be asked to come back for repeat testing? How many will be asked?<br>- Data analysis not addressed.<br>- No questionnaires included in attachments and only Borg CR10 scale specifically identified - qualitative elements of questionnaires that may be included not specified<br>- Location of study inconsistent throughout submission. Will football and running clubs be testing locations? The PIS only mentions university labs but there is a working offsite RA. Will any testing take place at football and running clubs as stated in G2? Any offsite testing will require documentation (letters of support, approvals, etc.).<br>- Recruiting strategy vague - no recruitment materials have been provided. If football or running clubs are to be contacted, how will this be done? What type of communication will go out over social media?<br>- If baseline testing will be conducted at one of two labs, wil there be other testing? The submission sometimes sounds lik there will be follow up testing (longitudinal) and sometimes like this will be crosssectional.<br>- More information about sensors and use is required.<br>- A sample of tests has been indicated. Is this a complete list Using "e.g." could mean other tests will be performed.<br>No information provided on sample groups (n=6).

8/10/2021	Reviewer B	why are there 6 sample groups? this is not clear, each group made up of 40 participants?<br><br>agree with all the comments from reviewer A,there are inconsistencies throughout the description. it is unclear if participants will be required to attend multiple times or once.<br><br>not clear which markers will be placed where and if collected during the same or different trials. These details need to be made much clearer before any ethical judgement can be made
8/20/2021	PI	This section has been substantially amended in line with the extensive comments from the reviewers.<br><br>- Sample size has been clarified and justifications have been added in G2 and M1. However, this is the first time that the DANU Sport wearable system has been used, so there is no previous studies or specific pilot data to based this on. Therefore we have used previous similar studies to based ou calculations on, as well as our own extensive literature review of the area, where differences have been seen in similar group sizes to our selected sample sizes.<br><br>-Sample groups stated more explicitly in G2 and M1. 'This project will involve two studies (Laboratory validation and Real-world Environment), that will involve two cohorts of 40 people (80 people in total). For study aims 1 and 2 will use the same participants that will be involved in the gait laboratory testing. Study aim 3 will involve a separate coho of participants.'<br>-Repeat testing now states 'All 40 participants will be asked to repeat the testing a week later for reliability purposes…'<br>- Outcome measures and data analyses now addressed in G2<br>-Pre-testing questionnaire (for researcher use only), SUS and RPE scale now included in G6<br>-Location of study clarified (i.e. Lab-based study and real-world environment), additional documentation now included in G6 .<br>-Recruitment strategy updated with in G2 and M1<br>- Recruitment materials such as contacts (and their relevant supporting documents) and recruitment flyer now attached i G6.<br>- Clarity on testing procedures evidenced in G2. Sub-study 1, participants will attend the lab 2x. Sub-study 2, researchers will attend the participants training location 5x (cross-sectional).<br>-Updated information on the sensors included (e.g. Sensor, components, locations to be worn)<br>- Location of wearable technology and markers clarified (e.g. Vicon Lower Limb Model).<br>-Protocol rephrased and updated
9/7/2021	Reviewer B	the extra information provides a clearer picture of the proposed methodology

Sample and Recruitment (see M1)

Add Save

Reviewer A: Approve

Reviewer B: Approve

e.g. Is the sampling approach appropriate to the design?
Is the sample sufficient and achievable?
Is the process of recruitment clearly explained?
Are participants receiving payments for taking part, and if so is the payment appropriate?
If the DBS is ticked, has the appropriate information been included?

DATE	ROLE	COMMENT
7/28/2021	Reviewer A	- No sample size indicated or justified.<br>- Indicated adults will be assessed over two years - unclear if crosssectional or longitudinal<br>- Will participants be asked to make sure they are not NHS staff? Is there a rationale for this?<br>- What are the 6 groups? How many participants for each?<br>- There is no mention of video ro images yet in the attachments such material can be used for various purposes including training. This must be clear, consistent and fully justified according to Northumbria regulations.<br>- Does the recruitment of sporting clubs outside of NE England involve them travelling to the university? The role o sports clubs is not clear from the submission.<br>- There will be recruitment via leaflets (not included in attachments and not mentioned above) and industrial sponso What role does the industrial sponsor have with recruiting and other aspects of the study. The sponsor's role needs to b explained if it is beyond funding.<br>- While risks of distress or discomfort may be extremely minimal, they cannot be completely eliminated.<br>- The university no longer has the U Drive so data cannot be stored there. Update data management information throughout accordingly.<br>- “The total time to complete this study will range according to which participant group they are in” - Provide more detai about this. G2 says approximately 2 hours.<br>- How many participants will come for a second visit? How will this be determined because it is dependent on group ?<br>- No risk assessments have been provided other than a Covid-19 RA and a travel risk RA. The activities involved require R and are likely covered by RAs in the RA library. Relevant RA need to be included in the ethics submission.
8/10/2021	Reviewer B	why are nhs staff excluded, reason for this not clear(funding issues?)<br><br>no sample size justification<br><br>why assessed 2years? looking for longitudinal differences?<br><br>do you need to make sure you have a mix of gender in each sample group to be generalisable?<br><br>previous or current injuries needs to be included in the exclusion criteria
8/20/2021	PI	-Sample size provided and justified in G2 and M1<br>-Sample groups stated more explicitly in G2 and M1<br>-The role of the sporting clubs now explicitly states that the researcher will travel to them<br>-NHS staff comment wasn’t justified therefore removed<br>-Video and photos now included and justified in G2 and M1<br>-Leaflet/poster (that will also be used for social media) now included in G6.<br>-Names for recruitment are now stated in M1 and letters of

		support are provided in supporting documentary evidence.<br>- The sponsor will not be directly involved with the study, any comments suggesting otherwise have been removed<br>- G2 and M1 now state 'All data will be anonymised, with th exception of optional video recordings where participants may give consent for their facial features to be recognisable in video or photos. The purpose of this would be for data checking (e.g. checking the videos if abnormalities are seen in the wearable sensor data), as well as staff protocol training, publications and conference presentations that will show the protocol being performed.'<br>-No risk of distress and discomfort has been changed to 'minimal and low'. Also stated what those risks are.<br>-Udrive has been consistently changed to OneDrive<br>-The time to complete protocol has been updated in G2.<br>-Inclusion criteria updated in M1 to state for Studies 1 and 2 '...an additional criteria of history of lower limb injury (that doesn't affect current participation in running activities) or no history of lower limb injury. This will be discussed when asking for medical history.'<br>- In regard to mix of gender the sample description (M1) states 'Participants regardless of their sex, sexuality, race will be involved in the study'. Gender is not a key metric from our study and can be controlled for within analysis if different between groups (i.e. entered as a co-variate), but we will endeavour to recruit equal numbers of male and female to attempt to control for this.<br><br>Sample size justifications have been added. However, this is the first time that the DANU Sport wearable system has been used, so there is no previous studies or specific pilot data to based this on. Therefore we have used previous similar studies to based our calculations on, as well as our own extensive literature review of the area, where differences have been seen in similar group sizes to our selected sample sizes.<br><br>There are two risk assessments; one for the laboratory testin and one for external travel / testing. Risk assessments have been provided in the documents section, which detail all risks for laboratory and external testing, including the research activities (e.g. exercise tests etc.) (not just COVID risks). This has been made clearer in the document; alterations are highlighted in yellow. These risk assessments have been extensively examined by the health and safety officers of the department (e.g. Amber Cassells) and approve for the full study details by the head of department prior to ethics submission, for all activities, not just COVID. However, we have also included relevant previous risk assessments fro the risk assessment library, as per the reviewer request, including treadmill use, electrical equipment use, on-campus testing, and off-campus testing / external institution testing.
9/7/2021	Reviewer B	the extra information provides sufficient information regarding the sample

Consent (see M1)

Add Save

Reviewer A: Approve

Reviewer B: Approve

e.g. Is the approach to consent seeking clear?
Is consent from parents/ carers/ guardians required?
Are all necessary recruitment and informed consent documentation included (e.g. letters of permission, letters of invitation)
Is the information sheet adequate to ensure informed consent?
Are the consent form(s) appropriate?

DATE	ROLE	COMMENT
**7/28/2021**	**Reviewer A**	**Two consent forms have been included in the attachments. There should only be one consent form. The longer consent form seeks permission to take videos and photos of participants. This is not mentioned in the main application. I photos and videos are to be taken the rationale must be clearly explained. It is not clear why these are necessary for training, publications, conferences, etc. from this application There is no explanation of keeping contact information o n file for future studies, especially if it is to be deleted after 12 months as indicated on the consent form above. There is no explanation of providing data to be accessed by other researchers via data depository. The longer consent form is beyond the normal and thus requires justification.**
**8/10/2021**	**Reviewer B**	**consent form states that participant data will be deleted aft 12months, but the protocol says the project will go on for 2 years...this is not clear<br><br>no mention prior to the consent forms of videoing / photos being included, if these part of the experimental design thes need to be mentioned in the research design**
**8/20/2021**	**PI**	**The two consent forms were included as one was an explicit COVID-19 exposure declaration, which is a separate form fro the study involvement consent form. This is standard practic from the department, as the health and safety officers wante separate consent for pandemic related outcomes. However, to meet the reviewers comment, we have condensed the COVID-19 form into the single consent form.<br><br>The study consent form contained standard items that are used for all of Dr Stuarts clinical and non-clinical studies.<br><br>Rationale for photos/videos updated in G2 and M1. We will collect this data to confirm any abnormal signals seen in the wearable sensor data. However, this is optional as participants can still be involved in the study without this. Similarly, we may use the videos to show other staff (for training on the protocol), or at conferences or within publications to show the protocol, but this is explicitly optional and participants can decline this, as well as decline being recognisable in the videos that may be used.<br><br>Typically, personal data (name, telephone number) is kept fo a period of 12 months, to ensure that researchers can contac participants if needed, even after their involvement. We provide an optional consent to be contacted about future studies, which would be possible within the 12month window of their contact information being available. This is standard on clinical research studies, and we are applying th same rigorous standards to this consent form.<br>Contact information comment removed from consent form<br><br>Consent form altered to state that study data (e.g. anonymou**

		signal and outcome data) will be stored, used and then deleted after 72 months, once the study has completely finished.<br><br>It is now standard practice for research studies to upload anonymous data to an online repository for other researcher to use in future, which would be done after the study has been completed (i.e. 72 months after the study finished). Th is good ethical practice for research data. There is no explici explanation for this, as it is a standard element of good research practice.
9/7/2021	Reviewer B	consent forms complete
9/14/2021	Reviewer A	See comments in the File Attachments section.

Researcher and Participant Safety (see M1)

Add Save

Reviewer A: Approve Reviewer B: Approve

e.g. Is there any risk of physical harm for the researcher(s) or the participants and if so what attempts have been made to alleviate or minimise them?
Have Risk Assessments been referred to where appropriate?

DATE	ROLE	COMMENT
7/28/2021	Reviewer A	- No risk assessments for activities have been provided. Thes must be included.<br>The travel risk assessment makes reference to "participants' homes". Why is this? The submission provides for testing in university facilities and any offsite testing needs to be detailed and is subject to all university approvals and documentation. The reason for this risk assessment is not clear, nor why entry into homes is necessary.
8/10/2021	Reviewer B	as per reviewer A can not conclude this as no risk assessmen has been included.<br><br>how will you ensure that the tasks are within their normal sporting activities? what speed will they run/ will this vary then between participants?<br><br>what if someone does injury themselves, what procedures ar in place?
8/10/2021	Reviewer B	risk assessment regarding COVID measures has been included, does not include risk assessments for the use of treadmill or wearable technology devices
8/20/2021	PI	There are two risk assessments; one for the laboratory testin and one for external travel / testing. Risk assessments have been provided in the documents section, which detail all risks for laboratory and external testing, including the activities (e.g. exercise tests) (not just COVID risks). These have been extensively examined by the health and safety officers of the department (e.g. Amber Cassells) and approve for the full study details by the head of department prior to ethics submission.<br><br>We have edited the risk assessments as per the reviewer comments;

		-Risk assessments have been altered (Item 8, 9 and 12 highlighted in yellow on general from and item 7 and 8 on travel form (below general form)) and approved by the department / supervisor<br>- Participants home removed, should have only stated their training location<br>-Protocol for injury has been added to the risk assessment (highlighted in yellow)<br>-Tasks will be within their normal sporting activities is ensured based on the inclusion criteria, more detail on the protocol has been provided in G2. 'Self-perceived slow, medium and fast' and '10, 12, 14km/hr')
9/7/2021	Reviewer B	risk assessments provided and clear
9/14/2021	Reviewer A	The Covid-19 risk assessments have been modified to include other aspects of the study beyond Covid-19 measures. There are several issues related to this that must be addressed:<br>- Covid-19 risk assessments are specific to Covid-19. If Covid-19 ceases to be a risk, the Covid-19 risk assessments will no longer apply. In this case, there would not be any applicable risk assessments associated with the study.<br>- Covid-19 risk assessments must be approved by the Head of Department (as a minimum), unlike other risk assessments. Any changes to Covid-19 risk assessments must be approved i the same manner. This has not been done since the sign-off date by Sam Stuart is prior to the original submission.<br>- Risk assessments for the activities that are to take place must be performed. Some of these have risk assessments that can be found in the risk assessment library (If you do not know how to access this, ask your supervisor.). If the risk assessment does not already contain an appropriate risk assessment for one or more of the study activities, risk assessment(s) will need to be created. Once created, any ne risk assessment will need to be submitted to Amber Cassells.
9/14/2021	Reviewer A	If you require guidance on preparing risk assessments or ensuring that the risk assessments correctly cover Covid-19 o the study activities, please ask Amber Cassells. She is available to answer any risk assessment or health & safety questions you may have.
9/20/2021	PI	Following discussion with the ethics lead, the risk assessments have been approved. I appreciate the feedback and will keep this is mind for future reference

Research Activities (see G2-G8, M1-M5, H1-H5)

Add Save

Reviewer A: Approve

Reviewer B: Approve

e.g. Are the research tasks described clearly?
Do sensitive topics such as trauma, bereavement, drug use, child abuse, pornography or extremism/ radicalism inform the research? If so have these been fully addressed? (and we can use this to amend the information on risk levels on the form)Is there any risk that the tasks may cause psychological harm and if so what attempts have been made to alleviate or minimise them?

DATE	ROLE	COMMENT
7/28/2021	Reviewer A	- A DBS clearance certificate number has been provided. The study is for non-vulnerable adults so the reason for this inclusion is not clear.

7/28/2021	Reviewer A	- Details for funder are presumed to be correct. The role of the funder is not clear if they are recruiting participants.
8/10/2021	Reviewer B	the research tasks are not clearly defined, these need to be made much clearer before an ethical judgement can be made
8/20/2021	PI	Although the study involved non-vulnerable adults, the DBS was included as it is good practice for any researcher workin with humans to have this in place. This is typically done for all of Dr Stuarts research team members.<br>Role of the funder has now been clarified. They are not providing recruitment, just technical support for the wearable equipment.<br><br>Research tasks have been amended in G2 to include more detail.
9/7/2021	Reviewer B	additional information enables ethical decision to be made, no issues

Data Management Plan (see G3)

Add Save

Reviewer A: Approve

Reviewer B: Approve

e.g. Have sufficient steps been taken to ensure participant anonymity/confidentiality of data?
Are the arrangements for data storage and disposal clearly outlined?
Are these arrangements in line with University and/or the funding body requirements?

DATE	ROLE	COMMENT
7/28/2021	Reviewer A	- The university no longer has a U Drive so data storage need to be updated accordingly.<br>- The time data will be stored is inconsistent, from 72 month to 10 years or potentially longer. This must be consistent an in accordance with Northumbria regulations.
8/10/2021	Reviewer B	in the consent form, one option is to agree to facial features being recognisable on video / photos but in data managemen plan it states all data will be anonymised
8/20/2021	PI	- Updated documents from U-drive to OneDrive<br>- Time data will be stored is now consistently stated as 72 months.<br>- Data management plan has been updated to state all data will be anonymised, except for videos where they may be recognisable if a participant provides consent to facial features being recognisable. Otherwise videos will not be collected.
9/7/2021	Reviewer B	data management plan sufficient

File Attachments (see G6)

Add Save

Reviewer A: Approve

Reviewer B: Approve

Please note: where file attachments have not been added because they are not required, please select Approve.

COMMENT BY	DATE	ROLE	COMMENT
	7/28/2021	Reviewer A	PIS

		- The aim of the study should reflect the study itself, not long-term goals. Individualised approaches to performance and sport injury assessment is beyond what can be determined by the proposed study.<br>- "We need to trial wearable technologies with as many participants as possible to ensure robust and confidence in results." This is not why someone should have been invited. It is also unethical to test more people than necessary to get the data necessary. Sample size needs to be provided and this should be deleted.<br>- The PIS says 18-55 years but G2 says 18-40 years This must be consistent.<br>- All information about what will be done is to be in the same section of the PIS. The figures should not be at the end. This should also be described s[illegible] that lay persons can understand in words. What is meant by "anthropometric data"? This needs to be explained for the potential participant. Provide some information of the walking, running, balance and jumping tasks (e.g. duration, intensity) so that the potential participant has the information necessary to make an informed decision.<br>- Wearing wearable devices is not a disadvantage of taking part in the study.<br>- Even is risks are low and minimal, they need to be stated. What are they?<br>- If a participant misses an appointment and you cannot contact them four times, contact details will be passed on to a medical practitioner - why is this? What is the reasoning behind this? What type of medical practitioner? This needs to be justified and align with Northumbria regulations.<br>- The university no longer has a U drive for data storage.<br>- The data storage needs to be consistent throughout.<br>- How will abnormalities bet determined that require the notification of the participant or health care provider. There is no indication elsewhere int he submission that any data can be used for diagnostic purposes. What categorises a risk of harm?<br>- The role of Danu needs to be clarified in the main application since their access to data and role with the study is not clear.<br>- What other organisation may access the data fro[illegible] the study?<br>- The PIS and debrief must include:<br><br>Name and contact details of the Records and Information Officer at Northumbria University: Duncan James (dp.officer@northumbria.ac.uk).<br><br>- Use consistent font size on PIS and debrief.
7/28/2021	Reviewer A	Debrief sheet<br><br>- Not being able to withdraw data because of data analysis having occurred is not a reason to not exclude data. Data should be able to be withdraw[illegible]

			until it has been published (i.e. when it is in the public domain).
	8/10/2021	Reviewer B	PIS outlines that various populations will be investigated, but nowhere else does it mention grouping populations, will different populations b[illegible] compared?<br><br>PIS does not outline how running gait will be analysed, what aspects of running gait? (stride length / frequency / joint positions?<br><br>PIS states it is an observational study, if collecting experimental measurements it is not purely observational. state that it is not a diagnosis exa[illegible] / test or the like.<br><br>PIS - no U drive, this needs to be altered. Is mentioned elsewhere also
rachel.mason	8/20/2021	PI	PIS<br>The participant information sheet has been substantially amended, based on the extensive reviewer comments.<br><br>- Aim has been altered and now reads 'This study aims to assess the validity and reliability of a will use novel wearable technology to objectively measure running gait, and then investigate the applied use of the technology in various populations'<br>- Sample size now provided (n=40)<br>- 18-55 years is now consistent across all documents<br>- Lay terms are now used and that section reformatted<br>- Low and minimal risks have been clarified<br>- If participant misses an appointment, has been removed from the document<br>- Data storage has been updated from U drive and is now consistent throughout<br>- Abnormalities/ Diagnostic purposes comment has been removed from the PIS<br>- Role of DANU clarified and their access to the anonymous data (e.g. signals from wearables).<br>- Stated that no other organisations apart from Northumbria University will have access to personal data (name, telephone number etc.)<br>- The aspects of running gait that will be analysed are now explicitly stated<br>Debrief<br>- Withdrawal of data has been rephrased to 'already could have been published'<br>General<br>- PIS and debrief now include Information Officer details<br>- Formatting across documents is now consistent

	9/7/2021	Reviewer B	file attachments approved
	9/14/2021	Reviewer A	The information and records officer ensures that Northumbria University applies laws relating to personal information of individuals. Duncan James does not have anything to do with withdrawing data from a study unless there is a potential legal issue or a participant has concerns about GDPR. This needs to be changed to correctly reflect his role.
	9/14/2021	Reviewer A	Debrief sheet - Not being able to withdraw data because of data analysis having occurred is not a reason to not exclude data. Data should be able to be withdrawn until it has been published (i.e. when it is in the public domain).
rachel.mason	9/20/2021	PI	Thank you for pointing this out, the PIS and debrief contact information have been updated to clarify DJ's role, also updated sentence regarding withdrawal of data (G6).

General Comments (see Help)

Add Save Help

DATE	ROLE	COMMENT
7/28/2021	Reviewer A	This ethics submission requires more detail to evaluate properly and the submission needs details to be consistent throughout since it is not possible to understand exactly what will be done.
8/10/2021	Reviewer B	the experimental design outlines soo far is not detailed enough or consistent enough to understand what the procedures are going to be.<br>There seems to be too many objectives to focus on for an initial investigation. Nowhere has the validity and reliabilit measurements been explored or explained. How is validity and reliability going to be established? I would recommend a narrow focus for the revised submission
8/20/2021	PI	- More detail has been added and inconsistencies have been addressed throughout<br>- Objectives have been narrowed down<br>- Exploration of validity and reliability of the device has bee addressed in G2: Research Activities<br><br>Thank you for the detailed feedback
9/7/2021	Reviewer B	the changes made have vastly improved the submission and a clear picture can be drawn of the proposed project now. I can't see any ethical issues with the project, happy to approve
9/14/2021	Reviewer A	The changes have made it possible to evaluate the submissio in regards to ethics. There are a few remaining things that need to be sorted, mainly concerning risk assessments.
