# Supplementary material for: Wearables for running gait analysis: A study protocol
Source: PLoS One. 2023 Sep 11;18(9):e0291289. doi: 10.1371/journal.pone.0291289 (PMC10495009; doi:10.1371/journal.pone.0291289)
Supplement: S1 File — (DOCX) [file pone.0291289.s004.docx]

**Wearables for Running Gait Analysis: A Study Protocol**

**Project Protocol Version 3.0**

**February 2022**

**INVESTIGATORS**

Rachel Mason

PhD Student

Department of Sport, Exercise and Rehabilitation, Northumbria University, Northumberland Building, Newcastle upon Tyne, NE1 8ST

e-mail: rachel.mason2@northumbria.ac.uk

Dr Alan Godfrey, PhD

Senior Lecturer

Department of Computer and Information Sciences, Northumbria University, UK

🕿 0191 237 3343

e-mail: [gill.barry@northumbria.ac.uk](mailto:gill.barry@northumbria.ac.uk)

Dr Gill Barry, PhD

Senior Lecturer

Department of Sport, Exercise and Rehabilitation, Northumbria University, Northumberland Building (NB318), Newcastle upon Tyne, NE1 8ST

🕿 0191 237 3343

e-mail: [gill.barry@northumbria.ac.uk](mailto:gill.barry@northumbria.ac.uk)

Dr Sam Stuart, PhD

Vice Chancellors Senior Research Fellow and Honorary Clinical Physiotherapist

Department of Neurology, Oregon Health and Science University, Portland, Oregon, USA

e-mail: samuel.g.stuart@gmail.com

# FUNDING AND SUPPORT IN KIND

| **FUNDER** | **FINANCIAL AND NON FINANCIALSUPPORT GIVEN** |
| --- | --- |
| **Northumbria University**  **DANU Sports Ltd, Dublin Ireland** | **PhD studentship**  **£36,000 (£12,000 per year for PhD Studentship).**  **In kind support equipment provided.** |

**Informed consent**

The participant must personally sign and date the latest approved version of the Informed Consent form before any study specific procedures are performed.

Written versions of the Participant information sheet and Informed Consent form will be verbally discussed and presented to the participants detailing no less than: the exact nature of the study; what it will involve for the participant; the implications and constraints of the protocol; any risks involved in taking part. It will be clearly stated that the participant is free to withdraw from the study at any time for any reason without prejudice to future care, without affecting their legal rights, and with no obligation to give the reason for withdrawal.

The participant will then be given time to consider the information, and will have the opportunity to question the Investigator, their GP or other independent parties to decide whether they will participate in the study.

Written Informed Consent will then be obtained by means of participant dated signature and dated signature of the person who presented and obtained the Informed Consent. The person who obtained the consent must be suitably qualified and experienced and have been authorised to do so by the Principal Investigator. A copy of the signed Informed Consent form will be given to the participant. The original signed form will be retained at the study site.

The informed consent form is presented below.

Objective Running Gait Measurement with Wearable Sensors for Sports Medicine and Performance Analysis

Name of researcher taking consent:

We are asking you to take part in a technology-based movement assessment related study as part of a PhD project.

Please be sure you have read the accompanying information sheet; it explains why we are doing this research and what we are asking you to do. If you find reading or understanding the information difficult, please ask a family member or friend to read it to you.

Please feel free to ask the research team any questions.

Remember, there is no obligation for you to take part in this research study. You are free to remove yourself from the study at any time.

Please contact the Chair of the Faculty of Health and Life Sciences Ethics Committee (Claire Thornton claire.thornton@northumbria.ac.uk) and state the full title and principal investigator of the study if you wish to make a complaint about the conduct of this study

Name of PhD Student: Rachel Mason

Email: Rachel2.Mason@northumbria.ac.uk

Name of Supervisor: Sam Stuart

Email: [Sam.Stuart@northumbria.ac.uk](about:blank)

Name of PhD Student: Fraser Young

Email: F.Young@northumbria.ac.uk

Name of Supervisor: Alan Godfrey

Email: Alan.Godfrey@northumbria.ac.uk

**Title:** Objective Running Gait Measurement with Wearable Sensors for Sports Medicine and Performance Analysis

|  | Initial | | | |
| --- | --- | --- | --- | --- |
| Please initial the boxes that apply to you | Consent | | Decline | |
| I confirm that I have read and understand the information sheet dated.............. for the above study. I have had the opportunity to consider the information, ask questions and have had these answered satisfactorily. |  | |  | |
| I understand that my participation is voluntary and that I am free to withdraw at any time without giving any reason, without my medical care or legal rights being affected. |  | |  | |
| I understand that individuals from this study research team at Northumbria University may look at my data collected during the study. |  | |  | |
| I give permission for my records / data to be used for this study. |  | |  | |
| I agree to anonymous movement data (including wearable technology data, videos and photographs) to be taken during my involvement within the study named above which may be during in a university setting (including club) or my own training environment, e.g. local sports club. |  | |  | |
| I understand that my personal data (e.g. name, address) will be stored at Northumbria University within secure facilities for 12 months, after which it will be disposed of. |  | |  | |
| I understand that all of my study data will be anonymised and stored at Northumbria University within secure facilities for 72 months, after which it will be disposed of. |  | |  | |
| I understand that all records are confidential and anonymous, and kept in locked filing cabinets which are accessed only by research staff working on this project. |  | |  | |
| I agree to participate in this study. |  | |  | |
| Additional (optional) studies |  | | | |
| I understand it will not be possible to connect my name to data taken for any future analysis for additional studies/work after it has been taken.  Any additional study may make use of the anonymous data collected from your involvement in this current study, i.e. your activity data from the activity monitors, your gender, age, weight, etc. These are classified as secondary analysis studies i.e. examining the data in different ways. This is done so anonymously, and you will not be contacted for any further information. All data is stored for 72 months after which it is disposed of if not regularly used. |  | |  | |
| I understand that the video and/or photographs will be stored on a secure computer under a participant study code (e.g. PD01) in the Clinical Gait laboratory, Northumbria University and will only leave the unit for the purposes agreed to below. I understand that my name and personal details will not be kept with the video and, as such, I cannot be identified from it. |  | |  | |
| I consent to my facial features being recognisable on video / photography. |  | |  | |
| I consent to the use of video / photographs collected on the study for  • internal staff training  • inclusion in presentations at national and international educational meetings  • inclusion in publications including academic journals and their internet sites |  | |  | |
| I agree to be contacted about ethically approved research studies for which I may be suitable. I understand that agreeing to be contacted does not oblige me to participate in any further studies. |  | |  | |
| I agree for my anonymised data to be archived in a suitable discipline data repository, and anonymised data may be used in future by other researchers. |  | |  | |
| COVID-19 | | | | |
| I have been made aware of the local site policies in respect of COVID-19. | |  | |  |
| I have completed and have returned the COVID-19 exposure declaration. | |  | |  |

**Name of researcher:** Rachel Mason

**Participant:**

Printed name: ________________________ Date: ________________________

Signed: _____________________________

**Parent / Guardian:**

Printed name: ________________________ Date: ________________________

Signed: _____________________________

**Person taking consent:**

Printed name: ________________________ Date: ________________________

Signed: _____________________________ Designation: __________________

**Settings and locations**

The setting for the study will be split between the biomechanics and clinical gait laboratory at City Campus and Coach Lane Campus, Northumbria University respectively (for laboratory testing) and a local park (for real-world environment testing).

The Biomechanics Laboratory and Clinical Gait Laboratory are dedicated facilities for the investigation of gait, balance, mobility and sports in healthy and clinical populations linking with the research themes of the Department of Sport, Exercise and Rehabilitation. The facility has all of the necessary equipment and space to allow this study to be conducted.

**Data Analysis**

The subjects or observations to be excluded, and the reason for their exclusion will be documented and approved by those responsible, prior to statistical analysis. Any exclusion documentation will be stored together with the remaining study documentation.

**Safety Considerations**

All measurements and interventions are non-invasive and place the subject at no risk other than those that normally may occur during standing, walking or running. For some of the participants, there is a slight possibility that they might feel some muscle soreness and fatigue after training. To prevent excessive fatigue, participants will be encouraged to take breaks as needed throughout all study procedures. A researcher from the study team will always be with the participant whilst the wearable technology is being used to monitor user-experience and ensure adequate rest periods are taken.

**Adverse Events:**

Any untoward medical occurrence, unintended disease or injury or any untoward clinical signs in participants whether or not related to the intervention will be recorded as an adverse event and managed according to the Health Research Authority (HRA) Guidance (see *below* for a description of adverse events taken from HRA Guidance)**.**

Any serious adverse event (SAE) occurring to a research participant will be reported to the main Research Ethics Committee if the event is related to the administration of the research procedures and unexpected.

Recording of adverse events will start from the signature of the informed consent until the participant has completed or passed through the Day 7 ± 1 days follow-up window. Information and assessments relating to the adverse event will be recorded in the adverse event section of the Case Report Form as per HRA Guidance and will be monitored until resolution or stabilization.

In the unlikely event of any serious adverse event occurring during this study, the University will be informed immediately (and no later than 72 hours after first awareness of the research team) and a written report provided. The university (acting as the Local Sponsor) will follow local reporting guidelines.

**Safety Reporting Procedures for Adverse Events**

**(Health Research Authority Guidance on Safety Reporting, Updated July 2020)**

An adverse event (AE) is any

- This includes events related to the intervention.

- This includes events related to the procedures involved (any procedure in the intervention).

Serious Adverse Events:

A Serious Adverse Event (SAE) is defined according to the health Research Authority (HRA) Guidance dated December 2010 as, any adverse event that:

- Results in death,
- Is life-threatening illness or injury, defined as an event in which the patient is at risk of death at the time of the event,
- Requires in-patient hospitalization or prolongation of existing in-patient hospitalization,
- Results in medical or surgical intervention to prevent life threatening illness or injury or permanent impairment to a body structure or a body function, or
- Results in persistent or significant disability[^*^](https://ukc-word-edit.officeapps.live.com/we/wordeditorframe.aspx?ui=en-us&rs=en-us&wopisrc=https%3A%2F%2Flivenorthumbriaac.sharepoint.com%2Fsites%2FWeeklyPhDSupervision%2F_vti_bin%2Fwopi.ashx%2Ffiles%2Fa7eb0e34f5244e38b01ca9b22aaa5fa3&wdenableroaming=1&mscc=1&hid=6446ae67-fcce-0cf1-45b8-5a4e6a48d61d-6877&uiembed=1&uih=teams&hhdr=1&dchat=1&sc=%7B%22pmo%22%3A%22https%3A%2F%2Fteams.microsoft.com%22%2C%22pmshare%22%3Atrue%2C%22surl%22%3A%22%22%2C%22curl%22%3A%22%22%2C%22vurl%22%3A%22%22%2C%22eurl%22%3A%22https%3A%2F%2Fteams.microsoft.com%2Ffiles%2Fapps%2Fcom.microsoft.teams.files%2Ffiles%2F98076398%2Fopen%3Fagent%3Dpostmessage%26objectUrl%3Dhttps%253A%252F%252Flivenorthumbriaac.sharepoint.com%252Fsites%252FWeeklyPhDSupervision%252FShared%2520Documents%252FGeneral%252FDRAFT%2520STUDY%2520PROTOCOL_3%2520-%2520November%25202020.docx%26fileId%3Da7eb0e34-f524-4e38-b01c-a9b22aaa5fa3%26fileType%3Ddocx%26ctx%3Dfiles%26scenarioId%3D6877%26locale%3Den-us%26theme%3Ddefault%26version%3D20201007007%26setting%3Dring.id%3Ageneral%26setting%3DcreatedTime%3A1605520901066%22%7D&wdorigin=TEAMS-ELECTRON.teams.files&wdhostclicktime=1605520900939&jsapi=1&jsapiver=v1&newsession=1&corrid=33476597-f915-44e5-b22e-bbbe030fa3e1&usid=33476597-f915-44e5-b22e-bbbe030fa3e1&sftc=1&sams=1&accloop=1&sdr=6&scnd=1&hbcv=1&htv=1&hodflp=1&instantedit=1&wopicomplete=1&wdredirectionreason=Unified_SingleFlush&rct=Medium&ctp=LeastProtected#_ftn1)/incapacity, or a permanent impairment of a body function or permanent damage to a body structure.

[^*^](https://ukc-word-edit.officeapps.live.com/we/wordeditorframe.aspx?ui=en-us&rs=en-us&wopisrc=https%3A%2F%2Flivenorthumbriaac.sharepoint.com%2Fsites%2FWeeklyPhDSupervision%2F_vti_bin%2Fwopi.ashx%2Ffiles%2Fa7eb0e34f5244e38b01ca9b22aaa5fa3&wdenableroaming=1&mscc=1&hid=6446ae67-fcce-0cf1-45b8-5a4e6a48d61d-6877&uiembed=1&uih=teams&hhdr=1&dchat=1&sc=%7B%22pmo%22%3A%22https%3A%2F%2Fteams.microsoft.com%22%2C%22pmshare%22%3Atrue%2C%22surl%22%3A%22%22%2C%22curl%22%3A%22%22%2C%22vurl%22%3A%22%22%2C%22eurl%22%3A%22https%3A%2F%2Fteams.microsoft.com%2Ffiles%2Fapps%2Fcom.microsoft.teams.files%2Ffiles%2F98076398%2Fopen%3Fagent%3Dpostmessage%26objectUrl%3Dhttps%253A%252F%252Flivenorthumbriaac.sharepoint.com%252Fsites%252FWeeklyPhDSupervision%252FShared%2520Documents%252FGeneral%252FDRAFT%2520STUDY%2520PROTOCOL_3%2520-%2520November%25202020.docx%26fileId%3Da7eb0e34-f524-4e38-b01c-a9b22aaa5fa3%26fileType%3Ddocx%26ctx%3Dfiles%26scenarioId%3D6877%26locale%3Den-us%26theme%3Ddefault%26version%3D20201007007%26setting%3Dring.id%3Ageneral%26setting%3DcreatedTime%3A1605520901066%22%7D&wdorigin=TEAMS-ELECTRON.teams.files&wdhostclicktime=1605520900939&jsapi=1&jsapiver=v1&newsession=1&corrid=33476597-f915-44e5-b22e-bbbe030fa3e1&usid=33476597-f915-44e5-b22e-bbbe030fa3e1&sftc=1&sams=1&accloop=1&sdr=6&scnd=1&hbcv=1&htv=1&hodflp=1&instantedit=1&wopicomplete=1&wdredirectionreason=Unified_SingleFlush&rct=Medium&ctp=LeastProtected#_ftnref1)Disability is defined as a substantial disruption of a person’s ability to conduct normal life functions.

*Severity of an Adverse Event*

Mild Adverse Event

A mild adverse event is one that the symptoms are barely noticeable to the patient. It does not influence performance, require drug treatment or prevent the patient from carrying on with normal life activities.

Moderate Adverse Event

A moderate adverse event is one that the symptoms make the patient uncomfortable and causes some impairment to normal life activities. Treatment for symptom(s) may be required.

Severe Adverse Event

A severe event is one that the symptoms cause severe discomfort to the patient and the severity limits the patient’s normal life activities. Treatment of symptom(s) may be required.

Ref. [Safety reporting - Health Research Authority (hra.nhs.uk)](https://www.hra.nhs.uk/approvals-amendments/managing-your-approval/safety-reporting/)

**COVID-19**

This study will follow the most up to date UK Government, NHS and Northumbria University Guidelines in all matters relating to Covid-19. This will include guidance on personal protective equipment (PPE) for both participants and researchers, and social distancing within the large gait lab space (which has an active ventilation system) and within an outdoor environment. The study involves the use of different wearable technologies and markers, which requires the researcher to be in close proximity. Only two researchers will be present during testing sessions in the Gait Lab to reduce the number of people in the room, and where possible carers or spouses who attend with subjects will be asked to wait outside in the seating area. All participants (and carers or spouses) will be asked if they have had any COVID-19 symptoms within the past several weeks over the phone the day before the visit, and this will be confirmed again on arrival at the Gait Lab. Researchers will wear appropriate PPE during the assessment, and the subjects and researchers will wash their hands upon touching others, or entering or leaving the room (alcohol gel will be available throughout the visit to be used when necessary). The gait laboratory will undergo a ‘deep clean’ for 30 minutes after the participant has attended the laboratory.

For interventions in the outdoor environment, researchers will adhere to Government Guidelines and local NHS protocols. A full risk assessment will be undertaken prior to commencing any interventions in the home environment which will include any measures relating to Covid-19 restrictions that are in place at the time of formulating the assessment such as social distancing guidelines and infection control measures.

**Data Protection and Patient Confidentiality**

The study will comply with the General Data Protection Regulation (GDPR) and Data Protection Act 2018, which require data to be de-identified as soon as it is practical to do so. The processing of the personal data of participants will be minimised by making use of a unique participant study number only on all study documents and any electronic database(s). All data samples collected as part of this study will be anonymised with participants being assigned a unique study number (e.g. WT01, WT02 etc.). All electronically stored data (e.g. videos) will be named using the individuals study number to ensure confidentiality. The only information we will retain for our database will be the age and sex of participants and whether they are a patient. We will keep one hard copy of the assessment in locked filing cabinets in the Clinical Gait Laboratory, Coach Lane, Northumbria University. This is the only place where we store any personal details like names and addresses. This information is kept locked away and is only available to people directly running the study. These people will treat the information in the strictest confidence. Dr Samuel Stuart, the Principal investigator of this study, is ultimately responsible for the protection of this information.

The Principal investigator (Dr Stuart) will ensure that this study is conducted in accordance with relevant regulations and with Good Clinical Practice. The results of any tests will be kept strictly confidential. This data can only be accessed directly by the development team on the application and will be securely password protected. The data will be kept and stored according to the university's regulations and will be destroyed as such when the study is complete. There is no personal or identifiable data stored in the application itself, as that would be a breach of data security. Information is also kept in accordance to GDPR and will be destroyed according to the appropriate timescales. Once the study has completed its main objectives, data will be stored for 10 years after which it will be disposed of.

**Data Sharing**

The dataset may be used for secondary analysis with the consent of the participants (on consent form), and all participant documentation will reflect the potential future use of these data in research. To maximise impact from this research study, following the end of the study anonymous data will be securely stored on an external online repository, such as FigSHARE with which Northumbria University has an existing partnership. Secondary researchers will be required to reference the data being used.

**Access to Data**

Direct access will be granted to authorised representatives from the host institution for monitoring and/or audit of the study to ensure compliance with regulations. The anonymous dataset may be used for secondary analysis with the consent of the participants (via consent form). All patient documentation will reflect the future use of these data in research.

**Project Management**

The study will be run by Dr Samuel Stuart (Principal Investigator) and researchers within his team. The Northumbria University team will be responsible for ensuring progress of the study in relation to administrative, clinical and academic issues. All published output from the study will acknowledge researchers involved.

**Insurance Indemnity**

The University has a specialist insurance policy in place which would operate in the event of any participant suffering harm as a result of their involvement in the research (U.M. Association Limited)**.**

**Peer Review**

This project and protocol design (including cohorts and statistical analysis) has been internally peer reviewed by the study investigators plus an independent expert from the University, with feedback incorporated into the study design and protocol.

### **Dissemination policy**

The data arising from the study is owned by Northumbria University:

On completion of the study, the data will be analysed and tabulated and a Final Study Report prepared.

The final study report can be accessed in the office of the PI, Dr Samuel Stuart.

- All participating investigators have rights to publish any of the study data, with agreement from the other investigators.
- The participants will be notified of the outcome of the study via a specifically designed newsletter.
- Participants can specifically request results which will be provided after the Final Study Report had been compiled.
- The study protocol, full study report, anonymised participant level dataset, and statistical code for generating the results will be made available on request.

**Authorship eligibility guidelines and any intended use of professional writers**

To warrant authorship on publications all study investigators will be examined for the following four authorship criteria:

- Substantial contributions to the conception or design of the work; or the acquisition, analysis, or interpretation of data for the work; AND
- Drafting the work or revising it critically for important intellectual content; AND
- Final approval of the version to be published; AND
- Agreement to be accountable for all aspects of the work in ensuring that questions related to the accuracy or integrity of any part of the work are appropriately investigated and resolved.
